# Click Chemistry Selectively Activates an Auristatin Protodrug with either Intratumoral or Systemic Tumor-Targeting Agents

**DOI:** 10.1021/acscentsci.3c00365

**Published:** 2023-06-22

**Authors:** Jesse M. McFarland, Maša Alečković, George Coricor, Sangeetha Srinivasan, Matthew Tso, John Lee, Tri-Hung Nguyen, José M. Mejía Oneto

**Affiliations:** Shasqi Inc., 665 3rd St, Suite 501, San Francisco, California 94107, United States

## Abstract

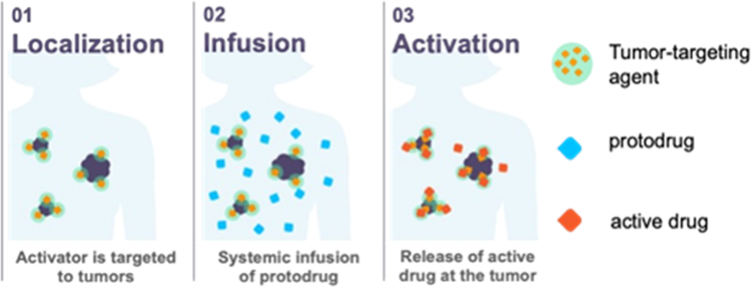

The Click Activated Protodrugs Against Cancer (CAPAC) platform enables the activation of powerful cancer drugs at tumors. CAPAC utilizes a click chemistry reaction between tetrazine and *trans*-cyclooctene. The reaction between activator, linked to a tumor-targeting agent, and protodrug leads to the targeted activation of the drug. Here, tumor targeting is achieved by intratumoral injection of a tetrazine-modified hyaluronate (SQL70) or by infusion of a tetrazine-modified HER2-targeting antigen-binding fragment (SQT01). Monomethyl auristatin E (a cytotoxin hindered in its clinical use by severe toxicity) was modified with a *trans*-cyclooctene to form the protodrug SQP22, which reduced its cytotoxicity *in vitro* and *in vivo*. Treatment of SQP22 paired with SQL70 demonstrated antitumor effects in Karpas 299 and RENCA murine tumor models, establishing the requirement of click chemistry for protodrug activation. SQP22 paired with SQT01 induced antitumor effects in the HER2-positive NCI-N87 xenograft model, showing that tumor-targeted activation could be accomplished via systemic dosing. Observed toxicities were limited, with transient myelosuppression and moderate body weight loss detected. This study highlights the capabilities of the CAPAC platform by demonstrating the activity of SQP22 with two differentiated targeting approaches and underscores the power of click chemistry to precisely control the activation of drugs at tumors.

## Abstract 2

Preclinical experiments show that a novel protodrug, SQP22, is activated by click chemistry and delivers monomethyl auristatin E to tumors using a biopolymer or an antibody fragment as a targeting agent.

## Introduction

Click chemistry encompasses chemical reactions that are fast, efficient, and selective in complex environments.^1^ The 2022 Nobel Prize in Chemistry was awarded to Sharpless, Meldal, and Bertozzi in recognition of the transformative effect click and bioorthogonal chemistry have had in research and drug development.^2^ The Click Activated Protodrugs Against Cancer (CAPAC) platform developed by Shasqi, Inc. (San Francisco, CA) is an approach that uses chemistry to engineer biology and precisely control the activation of drugs at tumor sites.^3^ Specifically, the CAPAC platform uses the tetrazine ligation reaction between tetrazine and *trans*-cyclooctene,^4^ an exceptionally fast and specific click chemistry reaction compatible with biological environments.^5−11^

Cancer is a major challenge worldwide, with estimates of 20 million new cancer cases and 10 million cancer-related deaths reported in 2020.^12^ Advances in targeted therapies, including immunotherapies, biomarker-targeting therapies, and antibody drug conjugates (ADCs), have revolutionized the treatment of certain cancers.^13−15^ Unfortunately, only subsets of patients expressing sufficient levels of specific biomarkers benefit from targeted approaches.^13−18^ This leaves systemically administered cytotoxic chemotherapeutic agents as a standard of care for a wide variety of cancers, despite their severe and often dose-limiting adverse effects and narrow therapeutic windows.^19−21^ The objective of the CAPAC platform is to address the clear need for an effective alternative to activate cancer therapies at tumors in the body.

The first investigational therapy based on click chemistry in humans, SQ3370, consists of two components: a tetrazine-modified biopolymer targeting agent (SQL70 biopolymer), which is injected intratumorally, and a *trans*-cyclooctene-modified protodrug of doxorubicin (SQP33 protodrug), which is infused intravenously (IV).^22^ The efficient *in vivo* reaction between the tetrazine moiety of SQL70 and the *trans*-cyclooctene moiety of SQP33 activates the protodrug, leading to the release of active doxorubicin at the tumor site. Results from the dose escalation in a phase 1/2a, first-in-human, single-arm, open-label trial in advanced solid tumors (NCT04106492) showed that SQ3370 is safe, well tolerated, and induces a cytotoxic T-cell supportive tumor microenvironment,^23−25^ consistent with data from previously published animal studies.^22^

Monomethyl auristatin E (MMAE) is a synthetic analogue of the natural product dolastatin 10 and a potent antimitotic agent that inhibits tubulin polymerization.^26,27^ MMAE has 100–1000 times more potent antitumor activity than doxorubicin; however, its use has been hindered by severe toxicities, including myelosuppression and neuropathy.^28,29^ Researchers have investigated several approaches to precisely target MMAE to tumors as a way to reduce its toxicity, including developing peptide conjugates^28,30^ and ADCs.^15,31,32^ The vedotin payload developed by Seagen, Inc. (Bothell, WA) links MMAE to antibodies via a protease-cleavable linker. As a testament to the power of this approach, four vedotin ADCs have been approved by the US Food and Drug Administration (FDA) for cancer treatment: brentuximab vedotin,^33^ enfortumab vedotin-ejfv,^34^ polatuzumab vedotin-piiq,^35^ and tisotumab vedotin-tftv,^36^ validating the benefits of the MMAE payload in oncology. These and other ADCs have demonstrated remarkable clinical benefits by directly targeting cancer cells via extracellular antigens to deliver a variety of payloads.^37^ To activate and release the therapeutic payload, internalization of the conjugate into tumor cells, followed by processing by endogenous proteases, is required. However, the requirement for internalization limits the scope of antigens amenable to targeting by ADCs as not all extracellular tumor-associated antigens are internalized.^38,39^ Moreover, many highly expressed antigens are secreted in the tumor stroma or located on cancer-associated fibroblasts rather than on the tumor cells themselves.^40^ A technology to activate MMAE specifically in the tumor microenvironment via noninternalizing or stromal cell antigens would complement ADC therapeutics.^40−42^ Other conditional activation strategies rely on physiological factors such as tumor-specific biomarkers, enzymatic activity, pH, or reactive oxygen levels to activate the payload based on differences between tumors and healthy tissues.^15,31,32,43−47^ The common characteristic of these approaches is that they rely on activation by preexisting biological parameters and thus are limited by inherent variations within a single tumor and across patients.

**Figure 1:**
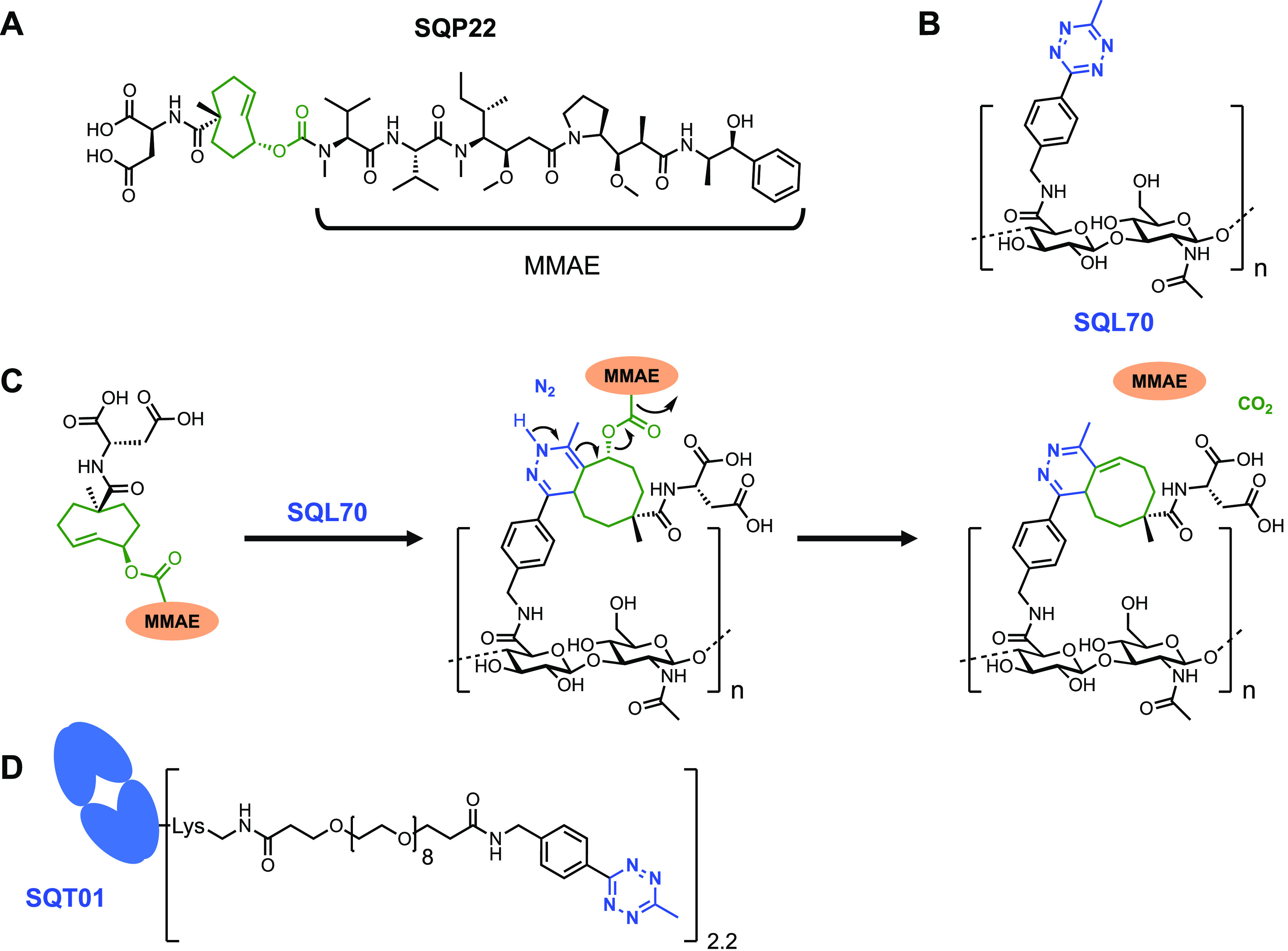
Activation of SQP22 protodrug by SQL70 biopolymer. (A) Chemical structure of SQP22, a TCO-modified protodrug of MMAE. (B) Chemical structure of Tz-modified biopolymer, SQL70. (C) Schematic of the click chemistry reaction by which SQP22 is activated by Tz on SQL70 biopolymer and active MMAE is released. (D) Chemical structure of Tz-modified Fab, SQT01. MMAE, monomethyl auristatin E; TCO, *trans*-cyclooctene; Tz, tetrazine.

The CAPAC platform, based on the tetrazine ligation reaction, was used to develop SQP22 (Figure 1A), a protodrug of MMAE, which is activated by tumor-targeting agents to release the MMAE payload specifically at tumors. Initially, we characterized SQP22 *in vitro* and *in vivo*, both alone and paired with the SQL70 biopolymer^22^ (Figure 1B). SQL70 is injected intratumorally and activates multiple systemically administered SQP22 protodrug doses to release MMAE into the tumor (Figure 1C). To explore the activation of SQP22 *in vivo* without tumor injections, we developed a human epidermal growth factor receptor 2 (HER2) antigen-binding fragment (Fab) conjugated to tetrazine and designated it as SQT01 (Figure 1D). By targeting a tumor-associated antigen, SQT01 can localize the tetrazine activators at the tumors that express the specific antigen. Desirable properties of the targeting antibody conjugate include: (1) tight binding to the tumor site with a slow off-rate and (2) rapid clearance from circulation to minimize systemic activation of the infused protodrug.

In this paper, we report the development of SQP22, an MMAE-based protodrug, and a companion tumor-targeting agent, the antibody-directed SQT01 conjugate. SQP22, when paired with either the intratumorally injected SQL70 biopolymer or SQT01, elicited sustained antitumor responses in the Karpas 299, RENCA, and NCI-N87 murine tumor models. Materials and methods for this article are provided in the Supporting Information.

## Results and Discussion

### SQP22 Design and Synthesis

MMAE was selected as the payload due to its successful use in FDA-approved ADCs.^33−36^ In each instance, MMAE is conjugated via a protease-cleavable linker,^33−36^ suggesting that release from the antibody is required to achieve full cytotoxic activity. As MMAE contains a secondary amine, it is an excellent candidate for modification with *trans*-cyclooctene. Inclusion of an aspartate residue on the *trans*-cyclooctene, as demonstrated with an etoposide-based protodrug,^3,48^ improved plasma stability and attenuation of *in vitro* cytotoxicity and yielded the protodrug, SQP22 (Figure 1A, Tables 1 and S1). A detailed description of the synthesis of SQP22 is included in the Supporting Information.

**Table 1:** *In vitro* Cytotoxicity of Activated SQP22^a^

	cytotoxicity IC_50_ (nM)		
cell line	+ tetrazine	– tetrazine	fold attenuation
MC38	4.6	3250	704
EMT6	2.8	1000	133
4T1	3.8	>200	>50
B16–F10	2.7	>200	>67
RENCA	1.8	>200	>100
NCI-N87	0.52	137	265
NCI-H460	2.9	448	157
A549	2.4	271	113

a Cytotoxicity of SQP22 protodrug after activation with tetrazine is shown as IC_50_ across several murine cancer cell lines. Cells were incubated with SQP22 with or without tetrazine activation for 72 h followed by analysis with CellTiter-Glo (Promega, WI). Fold attenuation was calculated based on the IC_50_ values in the presence of tetrazine (activated drug) relative to the absence of tetrazine (attenuated protodrug). IC_50_, half-maximal inhibitory concentration.

### SQP22 Cytotoxicity and Stability in Plasma and Tissue Homogenates

Modification of MMAE to form SQP22 protodrug reduced its cytotoxicity greater than 50-fold across several cell lines as assessed 72 h post drug treatment by CellTiter-Glo (Promega, WI) (Table 1). Activation of SQP22 by tetrazine activators led to the efficient release of MMAE and restored the activity of the drug with half-maximal inhibitory concentration (IC_50_) values in the 0.5–5 nM range, which was comparable to the potency of free MMAE (Table S1). SQP22 was highly stable in plasma, with approximately 94 and 100% of the protodrug remaining after a 4 h incubation period at 37 °C in human and mouse plasma, respectively (Table S2). Although up to 50% loss of SQP22 was observed when incubated over 24 h in tissue homogenates at 37 °C, no MMAE was released, supporting its potential safety *in vivo* (Figure S1).

### *In Vivo* Antitumor Efficacy of SQP22 in Various Murine Tumor Models

#### SQP22 Leads to Complete Tumor Regression When Combined with SQL70 in the Karpas 299 Xenograft Model

**Figure 2:**
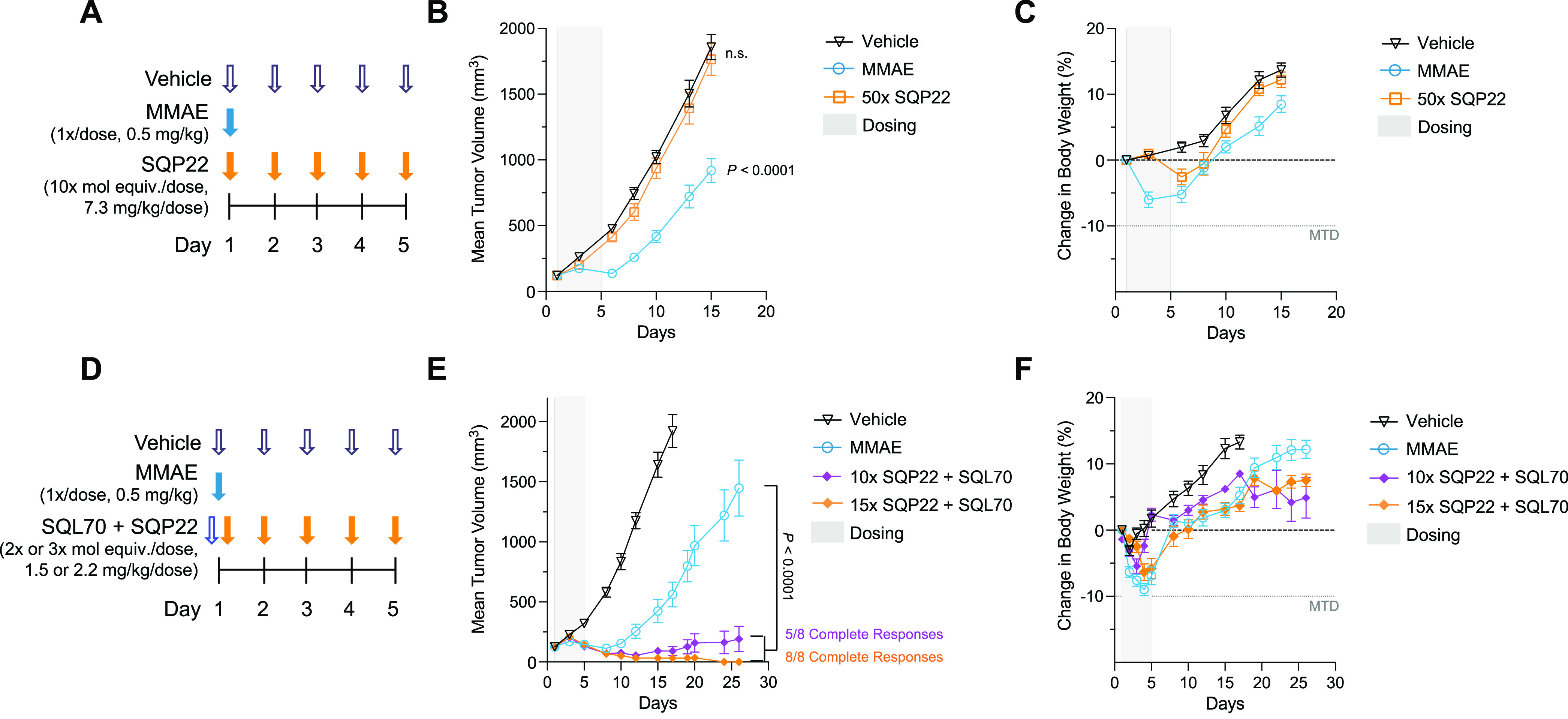
SQP22 leads to complete regression of Karpas 299 xenograft tumors in the presence of SQL70. (A) Schedule of dosing of agents in the absence of SQL70 biopolymer. (B, C) Tumor volumes of Karpas 299 xenografts in C.B-17 SCID mice (B) and body weight change (C) after dosing with vehicle, MMAE, and SQP22 protodrug. Only MMAE led to significant body weight loss on day 15 (*P* < 0.0001). (D) Schedule of dosing of agents in the presence of SQL70. SQP22 was dosed 1 h after SQL70 injection. (E, F) Tumor volumes of Karpas 299 xenografts in C.B-17 SCID mice (E) and body weight change (F) after dosing with vehicle, MMAE, and SQP22 protodrug with the SQL70 biopolymer. Shown are mean ± SEM (*n* = 8 mice/group). *P*-values were determined by two-way ANOVA with Bonferroni correction for multiple comparisons. Complete response was defined as no palpable tumors measured in 3 consecutive days. ANOVA, analysis of variance; MMAE, monomethyl auristatin E; mol equiv., molar equivalent; MTD, maximum tolerated dose; SEM, standard error of mean.

The attenuated cytotoxicity of SQP22 alone and its antitumor activity in the presence of SQL70 biopolymer were evaluated in the Karpas 299 xenograft model (Figure 2). While active MMAE administered as a single dose at 0.5 mg/kg (1×) led to a 2-fold reduction in tumor burden compared with the vehicle control at day 15 (*P* < 0.0001), SQP22 in the absence of SQL70 biopolymer had no effect on tumor growth relative to the vehicle control (*P* = 0.62), even when administered as 5-daily doses of 10× molar equivalents of MMAE (50× cumulative dose) (Figure 2A,B). Furthermore, 1× MMAE resulted in about 6% body weight loss during dosing with body weights remaining significantly lower than those in the vehicle control group at day 15 (*P* = 0.0011) (Figure 2C). On the other hand, SQP22 administered alone at 50× molar equivalents of MMAE led to minimal, transient body weight loss after dosing (<3% on average) with no significant difference relative to the vehicle control at day 15 (*P* = 0.64), demonstrating efficient potency attenuation and safety of the protodrug.

When administered in the presence of SQL70 biopolymer (Figure 2D), SQP22 at cumulative doses of 10× and 15× led to improved antitumor efficacy compared with 1× MMAE treatment starting on day 10, with eventual complete tumor regression in 5 of 8 and 8 of 8 animals, respectively (Figures 2E and S2), suggesting dose-dependent effects of SQP22. MMAE alone did not lead to complete responses in tumor-bearing animals. This demonstrates significant enhancement in efficacy by SQP22 at either dose with SQL70 compared with MMAE treatment (day 26, *P* < 0.0001) (Figure 2E). Body weight loss in the MMAE, 10× SQP22, and 15× SQP22 treatment groups was comparable and transient after dosing initiation, with the 10× SQP22 group recovering faster than the other groups (Figure 2F). Starting on day 20, the body weight change diverged between the 10× and 15× SQP22 treatment groups. Notably, the body weight in the treated animals did not drop more than 10%, suggesting manageable treatment-induced toxicity.

#### SQP22 with SQL70 Inhibits RENCA Tumor Progression with Transient Effects on Complete Blood Count

**Figure 3:**
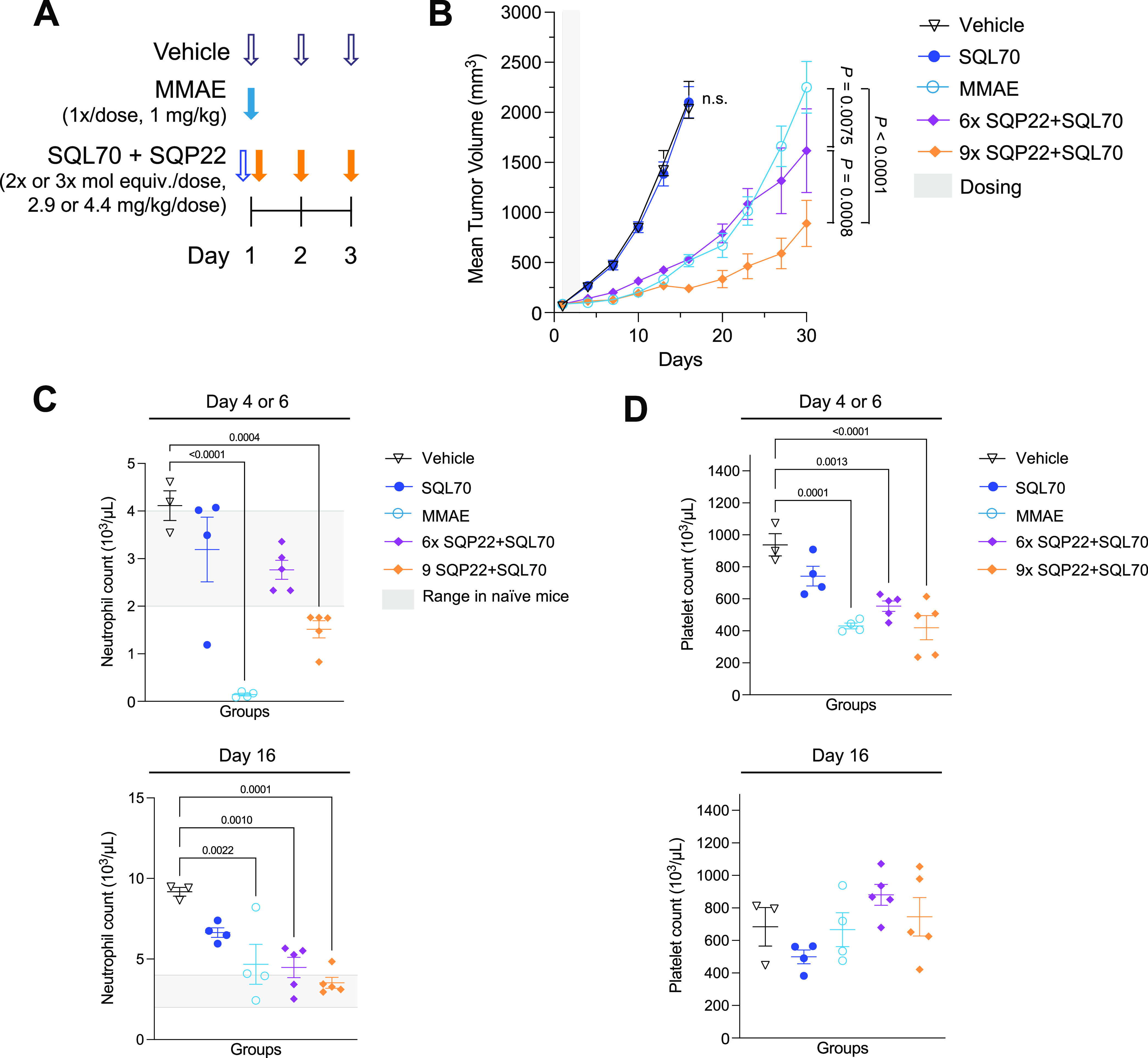
SQP22 with SQL70 reduces the growth of RENCA syngeneic tumors with reversible effects on complete blood counts. (A) Schedule of dosing of agents. SQP22 was dosed 1 h after SQL70 injection. (B) Tumor volumes of RENCA tumors in BALB/c mice following treatment with vehicle (*n* = 3 mice), MMAE (*n* = 4 mice), SQL70 alone (*n* = 4 mice), and SQL70 with SQP22 dosed at 2× (*n* = 5 mice) and 3× (*n* = 5 mice) mol equiv of MMAE/dose. (C, D) On the indicated days, blood samples were collected and analyzed for neutrophil (C) and platelet (D) counts. Range in neutrophil cell counts from naïve BALB/c mice is indicated by the gray box.^49^ Shown are mean ± SEM *P*-values were determined by two-way ANOVA with *post hoc* Bonferroni correction for day 30 (B) and one-way ANOVA with *post hoc* Bonferroni correction (C, D). ANOVA, analysis of variance; MMAE, monomethyl auristatin E; mol equiv, molar equivalent; SEM, standard error of mean.

The effect of SQP22 in combination with the targeting biopolymer SQL70 was next evaluated in the RENCA syngeneic tumor model (Figure 3A). MMAE treatment at 1 mg/kg (1×) resulted in the reduction of tumor growth with a 4-fold difference in tumor volumes at day 16 (Figure 3B, day 16, *P* < 0.0001), supporting the use of the RENCA model as an MMAE-sensitive syngeneic tumor model. Mice administered only SQL70 biopolymer showed no difference in tumor volume (Figure 3B) or body weight change compared to mice administered vehicle control (Figure S3A). However, administration of SQL70 followed by SQP22 as 3 daily doses of 2× or 3× molar equivalents of MMAE (6× or 9× cumulative dose, respectively) led to a significant reduction in tumor progression compared with administration of vehicle control (day 16, *P* < 0.0001 for both) or MMAE (day 30, *P* = 0.0075 and *P* < 0.0001, respectively) (Figure 3B), supporting the strong antitumor effects of treatment even at a reduced dosing schedule. In the presence of SQL70, SQP22 showed greater antitumor efficacy at 9× compared with the 6× cumulative MMAE molar equivalents dose (*P* = 0.0008), confirming a dose response over the study duration.

Of note, untreated RENCA-bearing mice lose body weight as tumors progress; hence, we evaluated the acute effects of treatments by analyzing the maximum body weight change in the first week after dosing initiation. No difference in acute body weight loss was observed between mice that were administered SQL70 or vehicle control (Figure S3B). Unlike MMAE treatment, which resulted in ≥15% body weight loss within the first 7 days after dosing, neither dose of SQP22 in the presence of SQL70 resulted in a significant reduction in body weight compared with vehicle control (Figure S3A,B). In fact, acute body weight loss in mice treated with SQP22 and SQL70 was <10%, consistent with results observed in the Karpas 299 model.

As MMAE has been shown to exhibit a myelosuppressive effect, we next evaluated acute and longer-term changes in the complete blood count of the RENCA-bearing mice. Blood samples drawn 3 days after the final dose (day 4 for the MMAE group, day 6 for all other treatment groups) and on day 16 were analyzed for complete blood counts (Figure 3C,D). On day 4, acute neutropenia was observed in MMAE-treated mice relative to vehicle (*P* < 0.0001), while moderate (∼50%) neutrophil reduction was observed on day 6 in mice treated with SQP22 at the cumulative dose of 9× molar equivalents of MMAE in the presence of SQL70 relative to vehicle control (*P =* 0.0004). Neutrophils for both groups were below the normal range reported for wild-type BALB/c mice (i.e., 20–30% of white blood cell count).^49^ On the other hand, mice receiving the cumulative dose of 6× mol equiv of MMAE displayed neutrophil counts within the normal range with no significant difference compared with the vehicle control group (*P* = 0.0653). Reductions in platelet counts to similar levels were measured in mice dosed with MMAE alone (*P* = 0.0001) and SQL70 with SQP22 at 6× (*P* = 0.0013) or 9× (*P* < 0.0001) cumulative doses (Figure 3D). Both acute effects observed, neutropenia and thrombocytopenia, were reversible by day 16 (Figure 3C,D), indicating transient MMAE-associated myelosuppression.

#### SQP22 in Combination with SQT01 Inhibits Tumor Progression in the HER2-Positive NCI-N87 Xenograft Model

SQT01 was designed as a Fab-tetrazine conjugate to balance the need to specifically bind the HER2 antigen with high affinity without the extended circulation associated with a full IgG. Generation and characterization of SQT01 are presented in the Supporting Information (Materials and methods, Figures S4A,E and S5A,B). Briefly, the purified Fab of trastuzumab was conjugated on lysine residues with tetrazine-PEG9-NHS and the resulting tetrazine-to-antibody ratio was determined to be 2.2 (Figure S4C). The conjugate was highly monomeric (Figure S4D) and bound antigen-positive cells similarly to the unconjugated Fab (Figure S5A,B). A nonbinding isotype control Fab-tetrazine conjugate was prepared and characterized by similar methods (Figure S6A–D).

**Figure 4:**
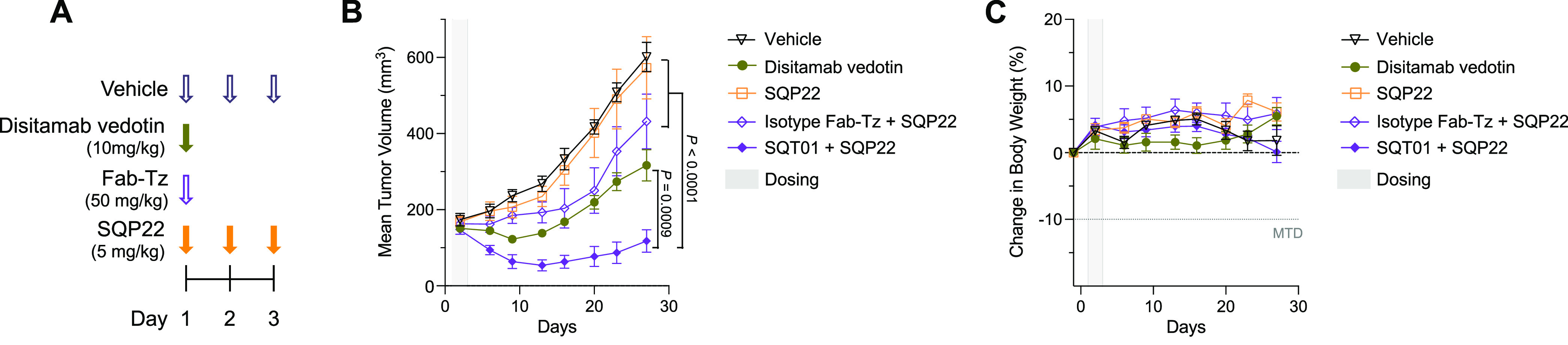
SQP22 leads to regression of NCI-N87 xenograft tumors in the presence of SQT01. (A) Schedule of dosing of agents. Two types of Fab-Tz agents were used: an isotype control and SQT01. On day 1, SQP22 was dosed 4 h after Fab-Tz infusion. (B, C) Tumor volumes of NCI-N87 xenografts in SCID mice (B) and body weight change (C) after dosing with vehicle, distimab vedotin, and SQP22 protodrug alone or with isotype or SQT01. Shown are mean ± SEM (*n* = 6 mice/group). *P*-values were determined by two-way ANOVA with Bonferroni correction for multiple comparisons on day 27. ANOVA, analysis of variance; Fab-Tz, Fab-tetrazine; Isotype Fab-Tz, Isotype Fab-tetrazine; MTD, maximum tolerated dose.

The effect of SQP22 in combination with SQT01 was evaluated in the NCI-N87 xenograft model (Figure 4A). Based on imaging studies performed with a similar conjugate, we designed the study to include multiple doses of SQP22 as we hypothesize SQT01 may be found at the tumor up to several days post dose.^50^ While tumor-bearing mice treated with SQP22 alone displayed no effect on tumor growth compared with those treated with vehicle (*P* > 0.99), infusion of SQT01 4 h prior to SQP22 treatment resulted in a significant, 5-fold reduction in tumor size compared with the vehicle controls on day 27 (Figure 4B, *P* < 0.0001). Moreover, SQP22 with SQT01 showed significant inhibition of tumor progression compared with SQP22 alone or SQP22 with the isotype control Fab-tetrazine conjugate (*P* < 0.0001). The minimal activity observed in the nonbinding control group may be due to a small amount of the conjugate remaining in circulation at the time of SQP22 infusion. Disitamab vedotin is a HER2-targeted vedotin ADC being studied in multiple late-stage clinical trials targeting HER2-positive solid tumors.^51−53^ It was included as a positive control to benchmark the treatment against an ADC carrying an MMAE payload and showed only a 2-fold reduction in tumor burden compared with vehicle at the study end point (*P* < 0.0001). In fact, SQP22 with SQT01 achieved a greater antitumor effect than disitamab vedotin in this experiment (day 27, *P* = 0.0009).

Little to no body weight loss was observed in the group treated with SQP22 and SQT01 compared with the group treated with vehicle control (*P* > 0.99), suggesting minimal nonspecific activation of SQP22 (Figure 4C). We also confirmed that treatment with SQT01 alone had no effect on tumor growth or body weight compared to vehicle treatment (Figure S7).

These results support the conclusion that binding to HER2 by SQT01 effectively localized tetrazine activators at the tumor and led to the release of MMAE. Variables such as dose level, timing, and frequency for SQT01 and SPQ22 are being evaluated to reach an optimal dose and schedule for administration.

## Conclusions

The data presented in this article together with our previously published work^22^ highlight the power of click chemistry. A single tumor-targeting agent (SQL70 biopolymer) has been shown to activate two different protodrugs: SQP33, with a doxorubicin payload,^22^ and now SQP22, with an MMAE payload. In addition, the results demonstrate how a single protodrug (SQP22) can be activated by two different tumor-targeting agents with distinct dosing methods (intratumoral injection for SQL70 and systemic infusion for SQT01). The biopolymer targeting agent makes the treatment agnostic to tumor type as well as inherent differences in tumor biology that can vary within a single tumor and even more from patient to patient. However, the application of intratumoral injections is limited to certain tumor types and the wide spectrum of patient characteristics motivates the development of alternative tumor-targeting strategies.^54,55^ In certain settings, tumor biology can be exploited for specific targeting of the payload, for example, by using SQT01 to target HER2-positive tumors such as those occurring in breast cancer, non-small-cell lung cancer, and gastric cancer. The treatment of solid tumors remains a significant challenge, and the two activating agents represent complementary targeting strategies.

The CAPAC platform is characterized by several features that unlock unique benefits. First, the use of click chemistry in humans differentiates this approach from biology-based conditional activation strategies. The activation of the protodrug is not dependent on biological factors but rather is based on the fast, specific, and efficient tetrazine ligation reaction. Nor is the activator required to be taken up by cells allowing noninternalizing or extracellular antigens found in the tumor microenvironment to be used for tumor targeting.^40,41^ In addition, the reliance on chemical reactivity rather than tumor-associated biological activity is expected to improve the translatability of a therapeutic from preclinical models to humans as click chemistry appears to be unaffected by interspecies differences. The consistent performance of the lead CAPAC asset, SQ3370, across species and in a Phase 1/2a clinical trial supports this hypothesis.^22−25^

Second, the targeting agent is decoupled from the payload. This creates a modular system that enables several beneficial attributes. To start, each component can be optimized for its specific task. The targeting agent can be given in sufficient doses to maximize the probability of saturating the desired antigens in the tumor or the tumor microenvironment without the liability of an attached payload. Additionally, the payload can be dosed to maximize its effect (e.g., doses at day 1, 2, or 3) as long as the targeting agent with tetrazine remains at the tumor.

**Figure 5:**
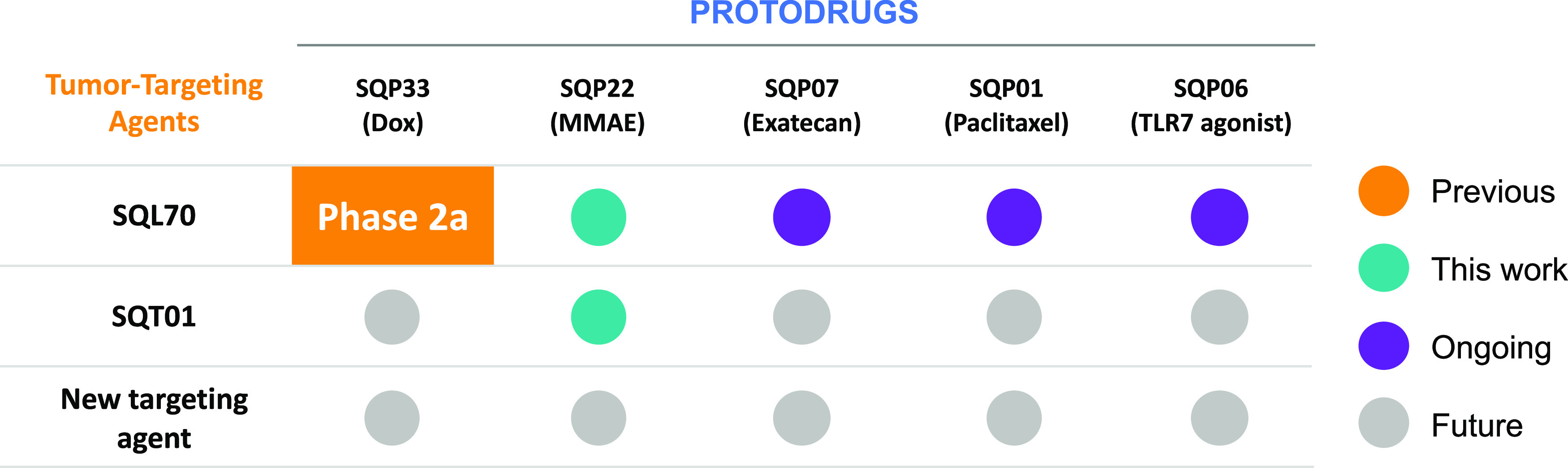
Flexibility of click chemistry for the targeted activation of drugs enables a modular platform in which a single targeting agent can be used with multiple protodrugs or vice versa. Thus, with the addition of each new protodrug or targeting agent, multiple new potential therapeutics are possible, each with the potential benefits unlocked by the platform.

Third, a targeting agent can activate any protodrug that has been previously created. Once a new tumor-targeting agent is created, it can be tested in the relevant models with an array of protodrugs, differing in, for example, potency or mechanisms of action, leading to critical insights in choosing a development candidate (Figure 5). Furthermore, the development path is simplified by using off-the-shelf protodrugs, which may have already been tested *in vivo* or even in clinical trials. As multiple protodrugs can be used with the same targeting agent, unique combinations and sequencing of therapies are possible that are currently prevented by overlapping toxicities.

Finally, the drug concentrations at the tumor achieved with our approach can unlock new biological effects. In the phase 1 clinical trial of SQ3370, the treatment has been reported to induce immune activation^24,25^ that may enhance a systemic antitumor response.^22^ Preliminary data with SQP22 and SQL70 have shown similar immune activation effects in the RENCA syngeneic murine model.^56^

Many questions still need to be addressed to further our understanding of the SQP22 protodrug and SQT01, both alone and in combination. Pharmacokinetic and biodistribution studies of SPQ22 and SQT01 will enable optimization of the dosing parameters, such as the dose levels, timing, and schedule. As the stoichiometry of the tetrazine ligation requires one activator for each protodrug molecule, understanding the efficiency of activator consumption will also guide the optimization of tetrazine loading and frequency of protodrug dosing. Ultimately, the translatability of the safety and efficacy of SQP22 with either SQL70 or SQT01 from animal models to humans has to be tested.

To summarize, we demonstrated the modularity and versatility of the CAPAC platform. Antitumor activity of the *trans*-cyclooctene-modified MMAE compound SQP22 in combination with either the intratumorally injected SQL70 biopolymer or the Fab-directed SQT01 conjugate was observed in multiple preclinical models. These results highlight the power of click chemistry to precisely control the activation of drugs at tumor sites using the tetrazine ligation reaction as well as support the hypothesis that this approach is agnostic to the format, molecular composition, or delivery method of the targeting agent or payload. Future work on investigational new drug application-enabling studies for SQP22, the development of new targeting agents, and the development of cytotoxic payloads (such as exatecan and paclitaxel^56^) as well as noncytotoxic payloads (such as immune agonists^56^) will further validate the CAPAC platform technology and its potential to engineer new characteristics for therapeutics within biological systems.

## Supporting Information Available

Materials and methods; synthetic procedures of SQP22 and the preparation of SQT01 and isotype conjugates; ^1^H NMR spectra; HPLC chromatograms; and flow cytometry and mass spectra (Figures S1–S19) (Tables S1 and S2) (PDF)
