# Supplementary material for: Click Chemistry Selectively Activates an Auristatin Protodrug with either Intratumoral or Systemic Tumor-Targeting Agents
Source: ACS Cent Sci. 2023 Jun 22;9(7):1400–8. doi: 10.1021/acscentsci.3c00365 (PMC10375897; doi:10.1021/acscentsci.3c00365)
Supplement: Supplementary file 1 — oc3c00365_si_001.pdf [file oc3c00365_si_001.pdf]

SUPPORTING INFORMATION

Click chemistry selectively activates an auristatin protodrug with either intratumoral or systemic tumor-targeting agents

Jesse M. McFarland, Maša Alečković, George Coricor, Sangeetha Srinivasan, Matthew Tso, John Lee, Tri-Hung Nguyen, and José M. Mejía Oneto*

jose@shasqi.com

Shasqi Inc., 665 3rd St, Suite 501, San Francisco, CA 94107, USA

**Table of Contents**	**Page number**
Materials and methods	S2-S15
**Table S1.** MMAE cytotoxicity in human and murine cancer cell lines	S16
**Table S2.** SQP22 stability in human and mouse plasma	S17
**Figure S1.** SQP22 protodrug stability in tissue homogenates	S18
**Figure S2.** SQP22 with SQL70 leads to complete regression of Karpas 299 tumors	S19
**Figure S3.** SQP22 with SQL70 leads to reduced body weight loss compared to MMAE	S20
**Figure S4.** Characterization of SQT01	S21
**Figure S5.** Characterization of SQT01 binding	S22
**Figure S6.** Characterization of Isotype Fab-Tz	S23
**Figure S7.** SQT01 has no effect on tumor volume or mouse body weight	S24
**Figure S8.** ^{1}H NMR of Compound **4**	S25
**Figure S9.** ^{1}H NMR of Compound **6**	S26
**Figure S10.** ESI-MS of Compound **6**	S27
**Figure S11.** ^{1}H NMR of Compound **7**	S28
**Figure S12.** ESI-MS of Compound 7	S29
**Figure S13.** ^{1}H NMR of Compound SQP22	S30
**Figure S14.** HPLC analysis of SQP22 (absorbance at 220 nm)	S31
**Figure S15.** ESI-MS of SQP22	S32
**Figure S16.** High resolution ESI-MS of SQP22	S33
**Figure S17.** HPLC analysis of MMAE (absorbance at 220 nm)	S34
**Figure S18.** Non-deconvoluted ESI-MS of (A) HER2 Fab and (B) SQT01 conjugate	S35
**Figure S19.** Non-deconvoluted ESI-MS of (A) Isotype Fab and (B) Isotype Fab-Tz conjugate	S36

Materials and methods

All chemicals were received from commercial sources and used without further purification unless otherwise noted. Column chromatography was performed with reagent grade silica gel (100-200 mesh) from Anhui Liangchen Silicon Material Co., Ltd (China). Analytical high performance liquid chromatography (HPLC) was performed with a Shimadzu LC-20AD fitted with a PDA detector using a Kinetex C18 5 µm analytical column (4.6 x 50 mm) using a gradient of CH_3CN (0.01875% TFA) in H_2O (0.0375% TFA) with a column temperature of 50 °C. Preparative HPLC was performed using a Phenomenex Luna C18 10 µm preparative column (200 x 40 mm) using a gradient of CH_3CN in H_2O (0.075% TFA). Electrospray ionization-liquid chromatography mass spectrometry (ESI-LCMS) was performed with a Shimadzu LCMS-2020 using a Kinetex EVO C18 5 µm analytical column (2.1 x 30 mm) using a gradient of CH_3CN (0.01875% TFA) in H_2O (0.0375% TFA) with a column temperature of 50 °C. Proton nuclear magnetic resonance (^{1}H NMR) spectroscopy was performed on a Bruker Avance NEO NMR at 400 MHz.

Safety statement: No unexpected or unusually high safety hazards were encountered in the execution of the experiments for this manuscript.

Preparation of SQP22 protodrug, SQL70 biopolymer, and the HER2 Fab-tetrazine conjugate SQT01

Synthesis of SQP22

The synthesis of the SQP22 protodrug is described in detail below (Scheme 1).

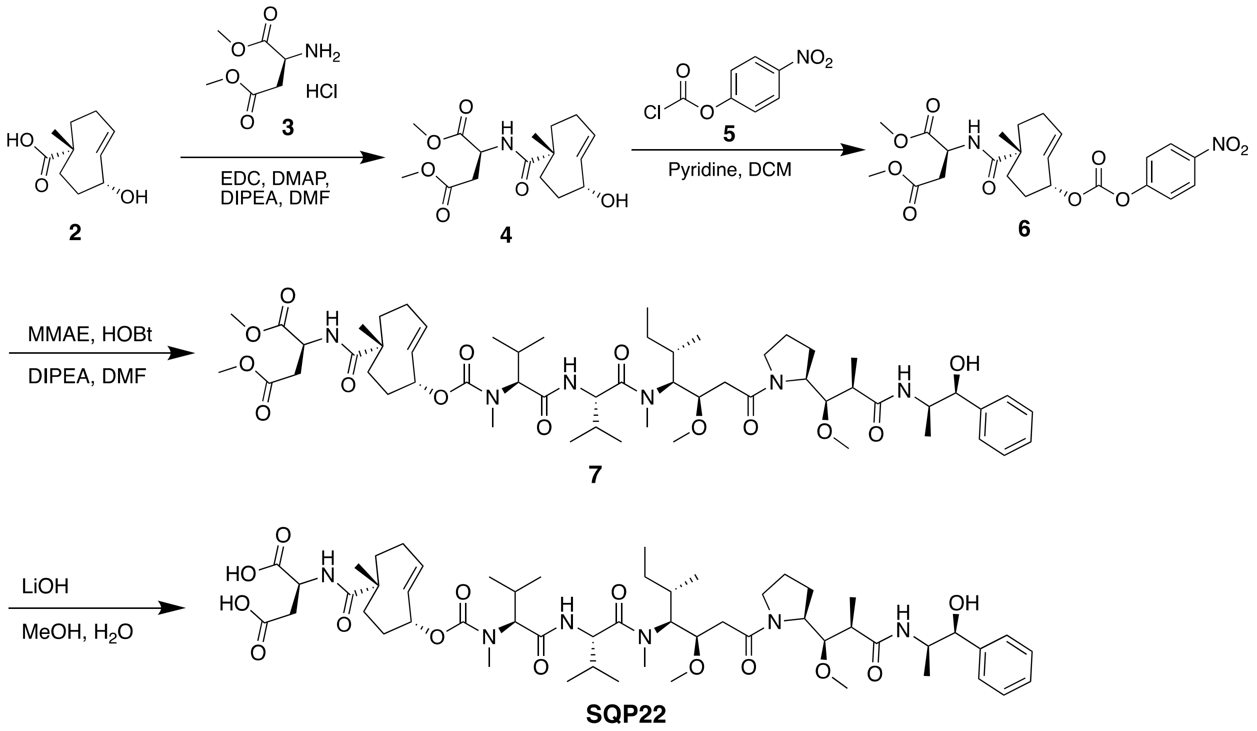

Scheme 1. Synthesis of SQP22 protodrug

*Preparation of compound 4***.** The steps for SQP22 synthesis are shown in Scheme 1. Compound **2** is synthesized using established methods.[1] To a solution of compound **2** (1.00 g, 5.43 mmol) in DMF (10 mL, dried overnight with 4A molecular sieve) was added DIPEA (2.10 g, 16.3 mmol, dried overnight with 4A molecular sieve), EDCI (2.08 g, 10.9 mmol) and DMAP (1.33 g, 10.9 mmol) and compound **3** (1.61 g, 8.14 mmol). The mixture was stirred at 25 °C for 16 hrs. TLC indicated compound **2** was consumed completely and one new spot formed. The reaction mixture was partitioned between DCM (20 mL) and H_2O (10 mL). The organic phase was separated, washed with saturated aqueous citric acid (3 mL) and brine (20 mL), then dried over Na_2SO_4, filtered, and concentrated under reduced pressure to give a residue. The residue was purified by column chromatography (Petroleum ether/Ethyl acetate = 3/1 to 1/1) and product containing fractions were evaporated under reduced pressure to give compound **4** (700 mg, 39.4% yield) as a white oil. ^{1}H NMR results of compound **4** are shown in Figure S8.

^{1}HNMR (400MHz, $CDCl_3$):

δ ppm 1.12 (s, 3 H), 1.60 (dd, *J* =15.45, 6.19 Hz, 1 H), 1.79 – 1.87 (m, 2 H), 1.92 (br d, *J* = 5.88 Hz, 1 H), 1.95 (s, 1 H), 1.98 – 2.00 (m, 1 H), 2.02 (br d, *J* = 4.13 Hz, 1 H), 2.26 (dd, *J* = 11.63, 3.88 Hz, 1 H), 2.30 – 2.36 (m, 1 H), 2.77 – 2.89 (m, 1 H), 2.88 – 2.88 (m, 1 H), 3.00 (dd, *J* = 16.95, 4.57 Hz, 1 H), 3.70 (s, 4 H), 3.75 (s, 3 H), 4.80 (dt, *J* = 8.00, 4.50 Hz, 1 H), 5.66 (dd, *J* = 16.63, 2.38 Hz, 1 H), 6.02 – 6.12 (m, 1 H), 6.54 (br d, *J* = 7.88 Hz, 2 H).

Preparation of Compound 6: To a solution of compound **4** (700 mg, 2.14 mmol) in DCM (5 mL) was added pyridine (846 mg, 10.7 mmol) and compound **5** (1.72 g, 8.55 mmol) in DCM (5 mL) (DCM was dried overnight with 4A molecular sieve). The mixture was stirred at 25 °C for 1 hrs. TLC indicated compound **4** was consumed completely and one new spot formed. The reaction mixture was partitioned between DCM (20 mL) and H_2O (10 mL). The organic phase was separated, washed with saturated aqueous citric acid (3 mL) and brine (20 mL), dried over Na_2SO_4, filtered, and concentrated under reduced pressure to give a residue. The residue was purified by column chromatography (Petroleum ether/Ethyl acetate = 3/1 to 1/1) to give compound **6** (490 mg, 46.5% yield) as a yellow oil. ESI-LCMS

calc $[M+H]^+$ 493.2; observe 493.2. ^{1}H NMR and ESI-MS results of compound **6** are shown in Figure S9 and Figure S10, respectively.

^{1}HNMR (400MHz, $CDCl_3$):

δ ppm 1.17 (s, 3 H), 1.55 – 1.61 (m, 1 H), 1.58 (br s, 1 H), 1.76 (dd, *J* = 14.76, 6.25 Hz, 1 H), 1.87 – 2.03 (m, 3 H), 2.06 – 2.15 (m, 1 H), 2.19 – 2.41 (m, 3 H), 2.82 (dd, *J* = 17.13, 4.50 Hz, 1 H), 3.03 (dd, *J* = 17.07, 4.44 Hz, 1 H), 3.72 (s, 3 H), 3.77 (s, 3 H), 4.78 – 4.86 (m, 1 H), 5.67 (dd, *J* = 16.70, 2.44 Hz, 1 H), 6.03 – 6.14 (m, 1 H), 6.58 (br d, *J* = 7.88 Hz, 1 H), 7.40 – 7.45 (m, 2 H), 8.27 – 8.33 (m, 2 H).

Preparation of compound 7: To a solution of compound **6** (490 mg, 995 μmol) and MMAE (714 mg, 995 μmol) in DMF (4 mL, dried overnight with 4A molecular sieve) was added DIPEA (64.3 mg, 497 μmol, dried overnight with 4A molecular sieve) and HOBt (202 mg, 1.49 mmol). The mixture was stirred at 25 °C for 16 hrs. LC-MS showed that compound **6** was consumed completely, and one main peak with desired mass was detected. The residue was purified by prep-HPLC and the product containing fractions were combined and lyophilized to give compound **7** (500 mg, 46.9% yield) as a white solid. ESI-LCMS calc $[M+H]^+$ 1071.7; observe 1071.6. ^{1}H NMR and ESI-MS results of compound **7** are shown in Figure S11 and Figure S12, respectively.

^{1}HNMR (400MHz, $CDCl_3$):

δ ppm 0.84 (br d, *J* = 6.75 Hz, 4 H), 0.89 (br d, *J* = 4.50 Hz, 5 H), 0.92 (br d, *J* = 6.63 Hz, 4 H), 0.98 (br d, *J* = 6.25 Hz, 3 H), 1.04 (br d, *J* = 6.88 Hz, 3 H), 1.16 (s, 3 H), 1.25 - 1.27 (m, 3 H), 1.59 - 1.74 (m, 3 H), 1.88 (br d, *J* = 9.38 Hz, 4 H), 2.07 (br d, *J* = 8.38 Hz, 5 H), 2.27 (br s, 4 H), 2.36 - 2.43 (m, 2 H), 2.45 - 2.53 (m, 1 H), 2.89 (br s, 6 H), 2.95 - 3.01 (m, 4 H), 3.04 (br s, 2 H), 3.29 - 3.34 (m, 3 H), 3.36 - 3.47 (m, 5 H), 3.67 - 3.73 (m, 4 H), 3.76 (s, 3 H), 3.82 - 3.89 (m, 1 H), 4.05 - 4.19 (m, 3 H), 4.28 (br s, 1 H), 4.63 - 4.86 (m, 3 H), 4.96 (d, *J* = 2.50 Hz, 1 H), 5.24 (br s, 1 H), 5.63 (br d, *J* = 18.14 Hz, 1 H), 5.82 (br s, 1 H), 6.53 - 6.74 (m, 3 H), 7.30 - 7.41 (m, 5 H).==

Preparation of SQP22: To a solution of compound **7** (500 mg, 0.47 mmol) in MeOH (5 mL) was added $LiOH \cdot H_2O$ (196 mg, 4.67 mmol) in H_2O (2 mL). The mixture was stirred at 25 °C for 16 hrs. LC-MS

showed compound **7** was consumed completely, and one main peak with desired mass was detected. The residue was adjusted pH ~ 2 with saturated aqueous citric acid, then purified by prep-HPLC and the product-containing fractions combined and lyophilized to give **SQP22** (265 mg 53.4% yield) as a white solid (HPLC purity 96.5% by peak area (RT 3.05 min) at 220 nm; MMAE not detectable, RT 2.11 min). HRMS calc $[C_{54}H_{87}N_6O_{14}{}^{+}, M+H]^{+}$ 1043.6275; observe 1043.6279. ^{1}H NMR, HPLC, and low-resolution and high-resolution ESI-MS results of compound SQP22 are shown in Figure S13, Figure S14, Figure S15, and Figure S16, respectively. HPLC results for MMAE are shown in Figure S17.

^{1}HNMR (400MHz, $CDCl_3$):

δ ppm 0.80 - 1.04 (m, 25 H), 1.09 (s, 3 H), 1.24 (d, *J* = 6.88 Hz, 3 H), 1.69 - 1.82 (m, 2 H), 1.88 - 1.94 (m, 3 H), 2.02 - 2.11 (m, 4 H), 2.16 (br d, *J* = 18.64 Hz, 1 H), 2.11 - 2.24 (m, 2 H), 2.32 (br d, *J* = 5.25 Hz, 2 H), 2.40 - 2.45 (m, 1 H), 2.52 (br d, *J* = 5.50 Hz, 2 H), 2.81 (br dd, *J* = 14.01, 4.88 Hz, 1 H), 2.94 - 3.04 (m, 2 H), 3.08 (s, 2 H), 3.14 - 3.27 (m, 6 H), 3.33 (s, 1 H), 3.39 (s, 3 H), 3.48 - 3.57 (m, 2 H), 3.94 (br d, *J* = 1.25 Hz, 1 H), 4.05 - 4.18 (m, 4 H), 4.30 (br dd, *J* = 6.19, 4.82 Hz, 2 H), 4.54 - 4.67 (m, 4 H), 4.91 (br d, *J* = 2.00 Hz, 2 H), 5.30 (br s, 1 H), 5.64 - 5.73 (m, 1 H), 5.79 - 5.89 (m, 1 H), 6.61 (br d, *J* = 7.38 Hz, 1 H), 7.30 - 7.42 (m, 5 H), 7.55 - 7.64 (m, 1 H).

Synthesis of SQL70

The synthesis of the SQL70 biopolymer has been described previously.[2]

Generation of the HER2 Fab-tetrazine conjugate SQT01

Trastuzumab Fab amino acid sequences:

Heavy chain:

EVQLVESGGGLVQPGGSLRLSCAASGFNIKDTYIHWVRQAPGKGLEWVARIYPTNGYTRYADSV
KGRFTISADTSKNTAYLQMNSLRAEDTAVYYCSRWGGDGFYAMDYWGQGTLVTVSSASTKGP
SVFPLAPSSKSTSGGTAALGCLVKDYFPEPVTVSWNSGALTSGVHTFPAVLQSSGLYSLSSVVTV
PSSSLGTQTYICNVNHKP SNTKVDKKV

Light chain:

DIQMTQSPSSLSASVGDRVTITCRASQDVNTAVAWYQQKPGKAPKLLIYSASFLYSGVPSRFSGS
RSGTDFTLTISSLQPEDFATYYCQQHYTTPPTFGQGTKVEIKRTVAAPSVFIFPPSDEQLKSGTASV
VCLLNNFYPREAKVQWKVDNALQSGNSQESVTEQDSKDSTYSLSSTLTLSKADYEKHKVYACE
VTHQGLSSPVTKSFNRGEC

Isotype Fab amino acid sequences:

Heavy chain:

EVQLLESGGGLVQPGGSLRLSCAASGFTFSSFSMSWVRQAPGKGLEWVSSISGSSGTTYYADSVK
GRFTISRDNSKNTLYLQMNSLRAEDTAVYYCAKPFPYFDYWGQGTLVTVSSASTKGPSVFPLAP
SSKSTSGGTAALGCLVKDYFPEPVTVSWNSGALTSGVHTFPAVLQSSGLYSLSSVVTVPSSSLGT
QTYICNVNHKPSNTKVDKKVEPKSSDKTHT

Light chain:

EIVLTQSPGTLSLSPGERATLSCRASQSVSSSFLAWYQQKPGQAPRLLIYYASSRATGIPDRFSGSG
SGTDFTLTISRLEPEDFAVYYCQQTGRIPPTFGQGTKVEIKRTVAAPSVFIFPPSDEQLKSGTASVV
CLLNNFYPREAKVQWKVDNALQSGNSQESVTEQDSKDSTYSLSSTLTLSKADYEKHKVYACEV
THQGLSSPCTKSFNRGEC

*Generation of SQT01***:** Coding sequences of the variable region of heavy chain and light chain of trastuzumab (human anti-HER2) antibody were used to generate HER2 Fab-expressing constructs. Coding sequences were synthesized and subcloned into PCDN3.4 expression vector. The constructed plasmids were transformed into *E. coli* for propagation and scale-up. Purified plasmids were confirmed by sequencing. The constructs containing the heavy chain and light chain of the HER2 Fab were transfected into HEK293 cells (suspension) with polymer polyethylenimine (PEI) reagent. The 6-liter culture medium

was harvested at 6-7 days post-transfection. The culture medium containing HER2 Fab was centrifuged, filtered, and then loaded onto KappaSelect affinity column (Mabselect Prism). The loading buffer was 25 mM Tris containing 150 mM NaCl, pH 8.0, and eluted with 100 mM sodium-citrate buffer containing 150 mM NaCl, pH 2.5. The collected solution was neutralized with 1 M arginine, 400 mM succinic acid buffer, pH 9.0. The affinity-purified protein was further purified by gel filtration with Superdex S-200 5/150GL column chromatography. The sample injection was 20 mL with a flow rate of 0.3 mL/min and mobile phase 2X PBS at pH 7.4. The purified HER2 Fab was analyzed by SDS-PAGE and SEC-HPLC. The final yield purified yield was 742 mg and stored in -80 °C for long term storage.

Generation of Isotype Fab-Tz: Coding sequences were synthesized and subcloned into pTT5 expression vector. The constructed plasmids were transformed into *E. coli* for propagation and scale-up. Purified plasmids were confirmed by sequencing. The constructs containing the heavy chain and light chain of the Isotype Fab were transfected into HEK293 cells (suspension) with polymer polyethylenimine (PEI) reagent. The 2-liter culture medium was harvested at 7 days post-transfection. The culture medium containing Isotype Fab was centrifuged, filtered, and then loaded onto KappaSelect affinity column (Mabselect Prism). The loading buffer was 25 mM Tris containing 150 mM NaCl, pH 8.0, and eluted with 50 mM sodium-citrate buffer containing 150 mM NaCl, pH 3. The collected solution was neutralized with 1 M arginine, 400 mM succinic acid buffer, pH 9.0. The affinity-purified protein was further purified by gel filtration with Superdex S-200 5/150GL column chromatography. The sample injection was 3 mL with a flow rate of 0.3 mL/min and mobile phase 2X PBS at pH 7.4. The purified Isotype Fab was analyzed by SDS-PAGE and SEC-HPLC. The final yield purified yield was 200 mg and stored in -80 °C for long term storage.

Tetrazine conjugation of HER2 Fab and Isotype Fab: HER2 Fab and Isotype Fab was buffer exchanged to PBS pH 7.4 overnight. The methyltetrazine-PEG9-NHS (SiChem #SC-8808) was dissolved in DMSO to make a 10 mM stock solution. For conjugation, the two components were reacted at 3:1 (methyltetrazine-PEG9-NHS to HER2 Fab) molar ratio at 25 °C for 2 hours. The amount of Fab for

tetrazine conjugation varied from 30-100 mg but the ratio of 3:1 was always maintained for each conjugation procedure. Then the solution was buffer exchanged to PBS pH 7.4 to remove excess methyltetrazine-PEG9-NHS and buffer salts.

Characterization of SQT01 and Isotype Fab-Tz: The sample of prepared SQT01 or Isotype Fab-Tz conjugate was analyzed by SDS-PAGE and Q Exactive HF-X LC-MS (Thermo Scientific) fitted with an Acquity UPLC protein BEH C4 column (300A, 1.7 µm, 2.1 x 50 mm) to confirm the formation of SQT01 conjugate and to determine the tetrazine-to-antibody ratio (Figure S4C, Figure S6B). HPLC-SEC analysis was performed using a UltiMate 3000 HPLC (Thermo Scientific) fitted with a XBridge BEH 200A SEC column (7.8 x 300 mm, Waters Corp). Proteins were eluted with PBS containing 15% isopropanol, pH 7.4). Size exclusion chromatograms for SQT01 and Isotype Fab-Tz and showed > 97% monomeric species for the Fab-tetrazine conjugate with minimal aggregation (≤ 3.0%) (Figure S4D, Figure S6C). The tetrazine-to-antibody ratio was calculated to be 2.2 for SQT01 (Figure S4C) and 1.8 for Isotype Fab-Tz (Figure S6B).

Flow cytometry analysis of SQT01 and Isotype Fab-Tz: Cell binding analysis by flow cytometry using NCI-N87 (HER2 positive) cells was tested with either unconjugated HER2 Fab, SQT01, or Isotype Fab-Tz control. NCI-N87 (HER2-positive) human gastric cancer cells were collected by centrifugation and resuspended with FACS buffer (PBS containing 2% FBS, pH 7.4). Cells were seeded in 96-well plates (200,000 cells per well) and centrifuged at 400 x g for 5 minutes. Supernatants were removed, and cells were incubated at 4 °C for 1 hour with 10-fold titration starting at 400 nM SQT01, HER2 Fab unconjugated, or isotype control (IgG) and for Isotype Fab-Tz cell binding experiment the titration was 3-fold starting at 300 nM. Plates were centrifuged, washed 3 times with FACS buffer and resuspended in 100 mL of secondary antibody of goat anti-human IgG Alexa 488 (catalog #A-11013, Thermo Fisher Scientific 1:500 dilution) or Goat anti-human (Fab') 2 fragment-specific (109-116-097, Jackson Immunoresearch, PA, USA, 1:200 dilution), and incubated in the dark at 4 °C for 1 hour. Supernatants were removed, cells washed twice with PBS and analyzed by CytoFLEX flow cytometer (Beckman

Coulter, Brea, CA, USA) or Attune NxT (Thermo Scientific, MA, USA). Mean fluorescent intensity (MFI) values were calculated and plotted against antibody concentrations. SQT01 binding to HER2-positive cells was observed to be comparable to unconjugated HER2 Fab (Figure S5A–B) and no binding was detected with Isotype Fab-Tz (Figure S6D).

In vitro assays to evaluate SQP22 cytotoxicity and stability in plasma and tissue homogenates

Cytotoxicity assay

The *in vitro* cytotoxicity of SQP22 in combination with methyltetrazine (Click Chemistry Tools, Scottsdale, AZ, USA, #1125) was tested in cancer cell lines (MC38, B6-F10, EMT6, RENCA) using a CellTiter-Glo (CTG) assay (Promega, Madison, WI, USA). MC38 and B16-F10 cells were cultured DMEM with 10% fetal bovine serum (FBS) and 1x penicillin-streptomycin (Pen/Strep), while EMT6 and RENCA were cultured RPMI 1640 supplemented with 10% FBS and 1x Pen/Strep. All cell lines were maintained in an incubator at 37 °C in an atmosphere of 5% CO_2. Cells at ~ 60-80% confluency were collected via trypsinization. One thousand MC38, B16-F10, EMT6 cells or two thousand RENCA cells in 95 µL of media were seeded into 96-well plates overnight. 10 mM SQP22 in DMSO was mixed with an equal volume of 10 mM tetrazine in DMSO or DMSO alone to generate 5 mM drug solutions of SQP22 or SQP22/tetrazine. These solutions were aged at ambient temperature for 15 minutes, protected from light, before being further diluted with complete media. Cells were treated at 8 concentrations generated via 5-fold serial dilutions (0.5 µM top concentration) and a non-treatment control. For MMAE, cells were treated at 8 concentrations generated via 3-fold serial dilutions (at either 0.1 or 0.03 µM top concentration). Cells were treated in triplicates for 72 hours before CTG assays.

The CTG assay was performed with the manufacturer's recommended procedure with modifications. Prior to the CTG assay, the plate and CTG reagent were at room temperature. CTG reagent (100 µL) was added to each well of the 96-well plate, and the contents were mixed to induce cell lysis. The plate's luminescent signal was stabilized for 10 minutes before reading by a microplate reader (FlexStation 3, Molecular Device, San Jose, CA, USA), and the signals were used to generate a viability

curve of drug concentrations versus cell response. The data was normalized to vehicle treated control wells. Non-linear regression curve was used to determine the IC_{50} of the parameters on the tested cells using GraphPad Prism 9 software.

Plasma stability assay

The metabolic stability of SQP22 was assessed in human and mouse plasma. The human and mouse plasma in K2 EDTA were obtained from BioIVT (Westbury, NY, USA). The assay was carried out in 96-well microtiter plates. Compounds were incubated in duplicate at 37 °C in the presence of plasma. Reaction mixtures (50 µL) contained a final concentration of 1 µM test SQP22. The extent of metabolism was calculated as the disappearance of the test compound, compared to the 0-minute control reaction incubations. Propantheline was included as a positive control to verify assay performance.

At each of the four time points, 300 µL of quench solution (50% acetonitrile, 50% methanol, and 0.05% formic acid, warmed up at 37 °C) containing internal standards was added to each well. Plates were sealed, vortexed, and centrifuged at 4 °C for 15 minutes at 4,000 rpm. The supernatant was transferred to fresh plates for LC-MS/MS analysis.

All samples were analyzed by LC-MS/MS using an AB Sciex API 4000 instrument coupled to a Shimadzu LC-20AD LC Pump system. Analytical samples were separated using a Waters Atlantis T3 dC18 reverse phase HPLC column (10 mm x 2.1 mm) at a flow rate of 0.5 mL/min. The mobile phase consisted of 0.1% formic acid in water (solvent A) and 0.1% formic acid in 100% acetonitrile (solvent B). MRM transitions for propantheline: *m/z* 368.3/181.1 and SQP22: *m/z* 1043.9/718.7.

The extent of metabolism was calculated as the disappearance of the test compound, compared to the 0-minute control reaction incubations. Initial rates were calculated for the compound concentration by plotting as a semi-log graph in GraphPad Prism and the slope was used to determine the time required for plasma concentration of the drug to decrease by 50% ($t_{1/2}$).[3] The $t_{1/2}$ for SQP22 was determined to be greater than 480 minutes in both species (Table S2). Propantheline was included as a positive control and was determined to have a $t_{1/2}$ of 0.68 hours in mouse plasma and 0.31 hours in human plasma.

Tissue homogenate stability assay

For sample preparation and processing, naïve mouse plasma, liver, spleen, heart, and kidney samples were obtained from BioIVT (Westbury, NY, USA). Two volumes of each tissue type in one volume of PBS (weight/volume) were homogenized by bead milling to prepare the homogenates. The samples were then spiked with SQP22 to a concentration of 1 µM and incubated at 37 °C. In duplicate, a 40 µL aliquot was collected at 0, 5, 15, 30 and 60 minutes, and at 2, 4, 8, and 24 hours. The samples, along with calibration samples with 0.5–1000 ng/mL of MMAE or SQP22, were processed by adding 4 volumes of ice-cold acetonitrile containing 100 ng/mL MMAF, and then centrifuged at 6,100 x g for 30 minutes. 170 µL of each supernatant was transferred to an autosampler plate insert and dried completely for about 2 hours on medium heat in the dryvac. The samples were then reconstituted with 20 µL of 2 mM ammonium acetate and 0.2% formic acid in water.

Sample analysis was performed using LC-MS/MS system, in which a Shimadzu VP Series HPLC system was in tandem with a SCIEX API 6500 (Foster City, CA, USA). The HPLC was equipped with a 20 x 2 mm Proto 200 C18 column (Higgins Analytical, Mountain View, CA, USA). An injection volume of 12.0 µL was used with a flow rate of 1.2 mL/min, and a gradient mobile phase A (2 mM ammonium acetate in water with 0.2% formic acid) and phase B (0.2% formic acid in acetonitrile). For detecting MMAE and SQP22 by mass spectrometry, a TurboIonSpray (ESI) in positive ionization mode was used with the cone voltage of 450 °C. Transitions monitored were used as follows: MMAE 718.6 –> 686.8 m/z; SQP22: 1043.6 –> 718.5 m/z; MMAF (as internal standard, IS) 732.6 –> 170.3 m/z. To calculate for the analyte concentration, the analyte peak area was normalized against the IS peak area. This calculation was performed for both the standards and unknown samples, and the resulting analyte concentration was reported in ng/mL.

Animal studies

All procedures related to animal handling, care, and treatment in the study were performed according to the guidance of the Association for Assessment and Accreditation of Laboratory Animal Care

(AAALAC) and approved by the Institutional Animal Care and Use Committee (IACUC) of WuXi AppTec (Nantong, China) or Cephrim Biosciences, Inc.

Karpas 299 xenograft model

The murine Karpas 299 (human non-Hodgkin′s large cell lymphoma) xenograft studies were conducted at WuXi AppTec. Karpas 299 cells were expanded in complete culture medium RPMI-1640 containing 20% FBS, 2 mM glutamine, and 1% Pen-Strep in a 37 °C incubator in an atmosphere of 5% CO_2. Cells were routinely passaged twice a week. Once the Karpas 299 cells reached the exponential growth phase, cells were harvested, washed, and counted for cell inoculation in mice. Female C.B-17 SCID mice 6-8 weeks old were inoculated subcutaneously at the right upper flank with 1 x 10^6 Karpas 299 cells in 0.2 mL of PBS with 50% Matrigel (1:1) for tumor development. Animals were distributed into groups of 8 for the efficacy study, and tumors were left to grow to group mean tumor volumes of ~100 mm^3.

To determine the attenuation of SQP22 *in vivo*, each group of 8 animals was administered vehicle control (10% hydroxypropyl-beta-cyclodextran [HPCD]) dosed IV on days 1–5; single IV dose of MMAE at 0.5 mg/kg (1x) on day 1; or SQP22 dosed at 7.3 mg/kg on days 1–5 (50-times [50x] molar equivalence of MMAE per dosing cycle) (Figure 2). To determine activation of SQP22 by the SQL70 biopolymer, each group of 8 animals was administered the vehicle control dosed IV on days 1–5; a single IV dose of MMAE at 0.5 mg/kg (1x) on day 1; SQP22 protodrug dosed at 2.2 mg/kg IV on days 1–5; a single dose of 100 µL of SQL70 biopolymer administered intratumorally followed by SQP22 protodrug dosed at 1.5 or 2.2 mg/kg IV 1 hour later on day 1 then dosed again on days 2, 3, 4, and 5 at 1.5 or 2.2 mg/kg (for 10x or 15x molar equivalents to MMAE [0.5 mg/kg] per dosing cycle of days 1–5) (Figure 2).

Tumor volume and body weight were measured two times weekly for both experiments. Tumor volume was measured using a caliper and calculated using the following formula: tumor volume (mm^3) = length x $width^2$ x 0.5. Complete response to treatment was defined as no palpable tumors measured over 3 consecutive days. Body weight loss was calculated according to the formula: percent

body weight loss = (starting weight minus current weight)/(starting weight) x 100. Animals were euthanized when tumors reached 2,000 mm^3 or body weight loss > 20% from the initial weight.

RENCA syngeneic model

The mouse RENCA (renal adenocarcinoma) syngeneic tumor studies were conducted at Cephrim Biosciences, Inc. RENCA cells were expanded in complete culture medium RPMI-1640 containing 10% FBS, 2 mM glutamine, and 1% Pen-Strep in a 37 °C incubator with 5% CO_2. Once RENCA cells reached the exponential growth phase, cells were harvested, washed, and counted for inoculation in mice. Female BALB/c mice 6-8 weeks old were injected subcutaneously at the right flank with 5 x10^5 cells in 100 µL of PBS. Animals were divided into groups of 3, 4 or 5 animals for the efficacy study, and tumors were left to grow to group mean tumor volumes of ~90 mm^3. The groups were administered vehicle control (10% HPCD) dosed IV on days 1–3, a single intratumoral dose of 40 µL of SQL70 biopolymer on day 1, a single IV dose of MMAE at 1 mg/kg (1x) on day 1, or SQL70 biopolymer dosed intratumorally on day 1 followed by IV dose of SQP22 one hour later at either 2.9 mg/kg or 4.4 mg/kg on days 1–3 (i.e., 6x, or 9x molar equivalents to MMAE [1 mg/kg] per dosing cycle, respectively) (Figure 3).

Tumor volume and body weight were measured three times weekly, with tumor volume and body weight change calculated as described for the Karpas 299 xenograft model above. Animals were euthanized when tumors reached 2,000 mm^3 or significant body weight loss of > 20% from the initial weight. For complete blood count, ~20 µL of whole blood was collected in EDTA tubes. Complete blood count was performed using the Drew Scientific HEMAVET Multispecies Hematology Analyzer according to the manufacturer’s instruction (Delta Scientific Inc., Miami Lakes, FL, USA).

NCI-N87 xenograft model

The NCI-N87 (gastric carcinoma) xenograft studies were conducted at Shanghai ChemPartner (Shanghai, China). NCI-N87 cells were cultured in RPMI-1640 supplemented with 10% FBS and 1% Pen-Strep in a 37 °C incubator with 5% CO_2. Cells were harvested and counted for tumor inoculation when the cells reached the exponential growth phase. Female C.B-17 SCID mice 6-8 weeks old were inoculated

subcutaneously at the right flank with 3 x 10^6 cells in a 200 µL mixture of RPMI-1640 with Matrigel (1:1) for tumor development. Animals were divided into groups of 6 for the efficacy study, and tumors were left to grow to tumor volume of ~100-150 mm^3 prior to dosing. All doses were given IV. Each group of 6 animals was administered the vehicle control (10% HPCD) on days 1–3; SQP22 alone at 5 mg/kg on days 1–3; a single dose of isotype Fab-tetrazine at 50 mg/kg on day 1 followed by SQP22 at 5 mg/kg on day 1 (4 hours after isotype conjugate) as well as on days 2 and 3; or a single dose of SQT01 at 50 mg/kg on day 1 followed by SQP22 at 5 mg/kg on day 1 (4 hours after the SQT01), as well as on days 2 and 3 (Figure 4). For a positive control group, animals received a single dose of disitamab-vedotin (Catalog # HY-P9985, MedChemExpress, Monmouth Junction, NJ, USA) at 10 mg/kg on day 1 (Figure 4).

Tumor volume was measured three times weekly in two dimensions using a caliper. Body weight was collected daily for the first week and twice weekly after the first week. Tumor volume and body weight change were calculated as previously described for the Karpas 299 xenograft model. Animals were euthanized when tumors reached 2,000 mm^3 or significant body weight loss of > 20% from the initial weight.

Statistical Analysis

Continuous variables are expressed as mean ± standard error of the mean (SEM), unless otherwise noted. For tumor volume and body weight changes, data were analyzed by two-way analysis of variance (ANOVA) with Bonferroni correction for multiple comparisons. For complete blood counts and maximum acute body weight change, data were analyzed using an ordinary one-way ANOVA with Bonferroni correction for multiple comparisons. In all cases, significance was defined as $P \leq 0.05$. Statistical analysis was carried out using GraphPad Prism 9 Software.

References

1. Wu, K., Yee, N. A., Srinivasan, S., Mahmoodi, A., Zakharian, M., Mejia Oneto, J. M. and Royzen, M. Click activated protodrugs against cancer increase the therapeutic potential of chemotherapy through local capture and activation. *Chem Sci*, 2021, *12*, 1259-1271.

2. Srinivasan, S., Yee, N. A., Wu, K., Zakharian, M., Mahmoodi, A., Royzen, M. and Oneto, J. M. M. SQ3370 activates cytotoxic drug via click chemistry at tumor and elicits sustained responses in injected & non-injected lesions. *Adv Ther (Weinh)*, 2021, *4*, 2000243.

3. Handbook of Basic Pharmacokinetics Including Clinical Applications, Sixth Edition: By Wolfgang A. Ritschel and Gregory L. Kearns Published by American Pharmacists Association, 2215 Constitution Avenue, NW, Washington, DC 20037, 2004. xii + 432 p.

Supplementary results

Table S1. MMAE cytotoxicity in human and murine cancer cell lines

Cell Line	MMAE IC_{50} (nM)
MC38	3.9
EMT6	ND
4T1	ND
B16-F10	0.16
RENCA	0.25
NCI-N87	0.30
NCI-H460	1.7
A549	1.2

MMAE cytotoxicity *in vitro* as assessed 72 hours post drug treatment by CellTiter-Glo (Promega, WI, USA). IC_{50}, half maximal inhibitory concentration; MMAE, monomethyl auristatin E; ND, not determined.

Table S2. SQP22 stability in human and mouse plasma

**Plasma Type**	**SQP22 Remaining After 4 Hour Incubation (%)**	**$t_{1/2,}$ (min)**
Human	94	>480
Mouse	100	>480

SQP22 protodrug was incubated in human or mouse plasma for 4 hours at 37 °C and the fraction remaining was quantified by LC-MS/MS. LC-MS, liquid chromatography with tandem mass spectrometry; $t_{1/2}$, time required for plasma concentration of the drug to decrease by 50%.

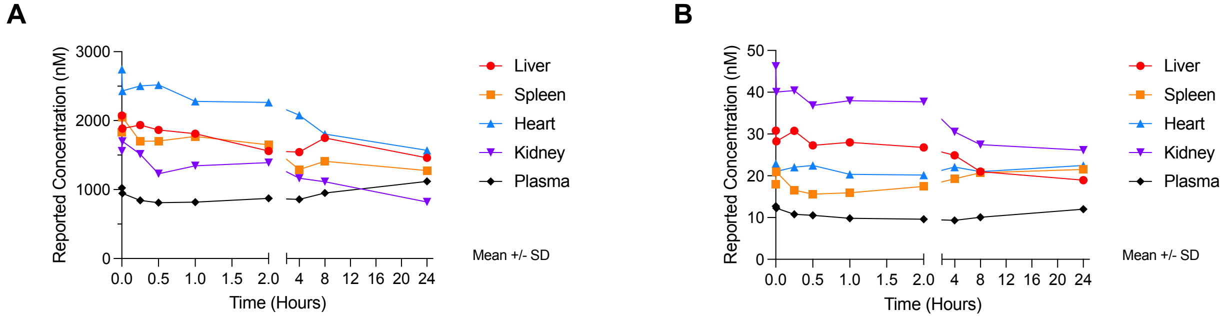

Figure S1. SQP22 protodrug stability in murine tissue homogenates. **A-B**, SQP22 was incubated at 37 °C in various tissue homogenates; samples were taken at the indicated time points and then analyzed for the concentration of SQP22 **(A)** and MMAE (**B**). Differences in apparent SQP22 starting concentrations are due to quantification with a standard curve prepared in plasma, rather than the matched tissue homogenate. MMAE present at time 0 likely represents a small amount of contaminant resulting from the synthesis of SQP22. Values shown are mean of replicates ± SD (*n* = 2 replicates/time point). MMAE, monomethyl auristatin E; SD, standard error.

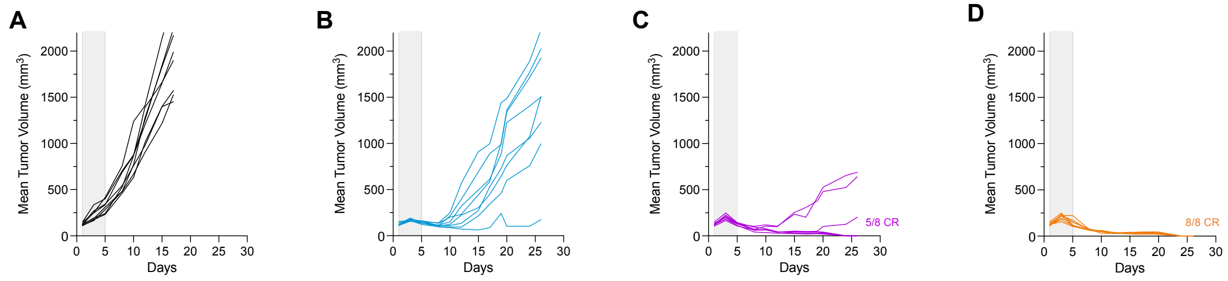

<u>Figure S2.</u> SQP22 with SQL70 leads to complete regression of Karpas 299 tumors. **A-D**, Individual tumor volumes in Karpas 299-bearing animals treated with vehicle (**A**), 0.5 mg/kg MMAE (**B**) or SQP22 at a cumulative dose of 10x (**C**) or 15x (**D**) molar equivalents of MMAE based on the dosing schedule described in Figure 2D.

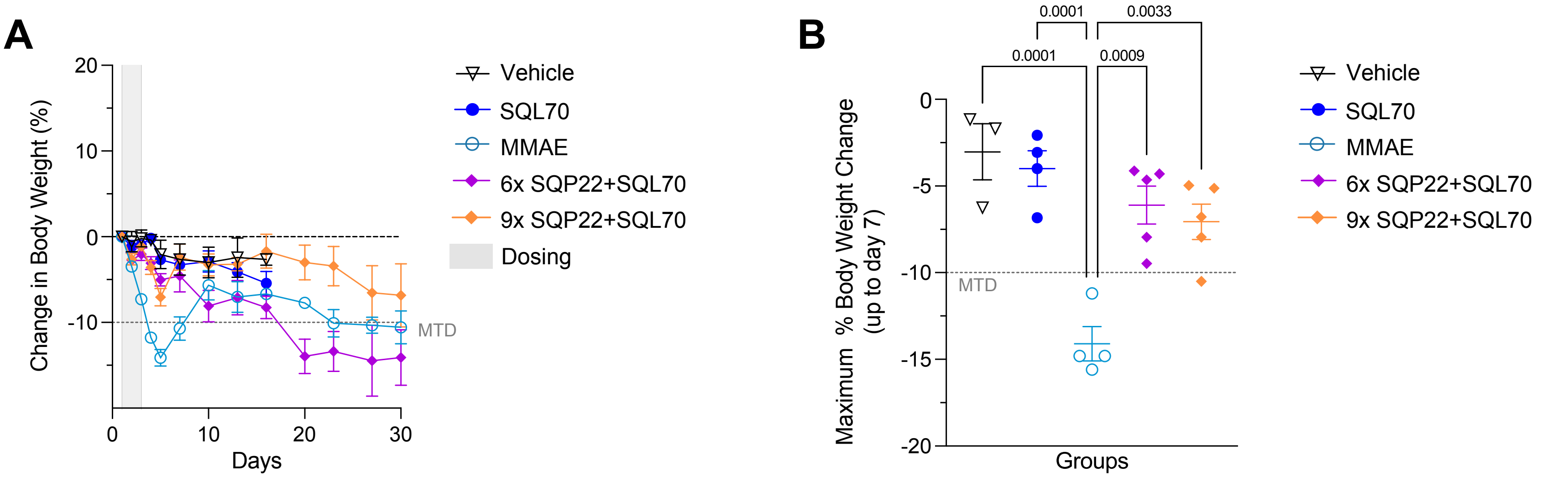

Figure S3. SQP22 with SQL70 leads to reduced body weight loss compared with MMAE. **A**, Body weight changes of BALB/c mice bearing RENCA tumors following treatment with vehicle (n = 3 mice), MMAE (n = 4 mice), SQL70 (n = 4 mice), and SQL70 with SQP22 dosed at 2x (n = 5 mice) and 3x (n = 5 mice) molar equivalents of MMAE/dose. **B**, Maximum body weight loss (shown as percentage) was assessed up to Day 7 to determine acute effects on body weight, since RENCA cells induce body weight loss as tumors grow. *P*-values were determined by one-way ANOVA with Bonferroni correction for multiple comparisons, compared to the vehicle group. ANOVA, analysis of variance; MMAE, monomethyl auristatin E; MTD, maximum tolerated dose.

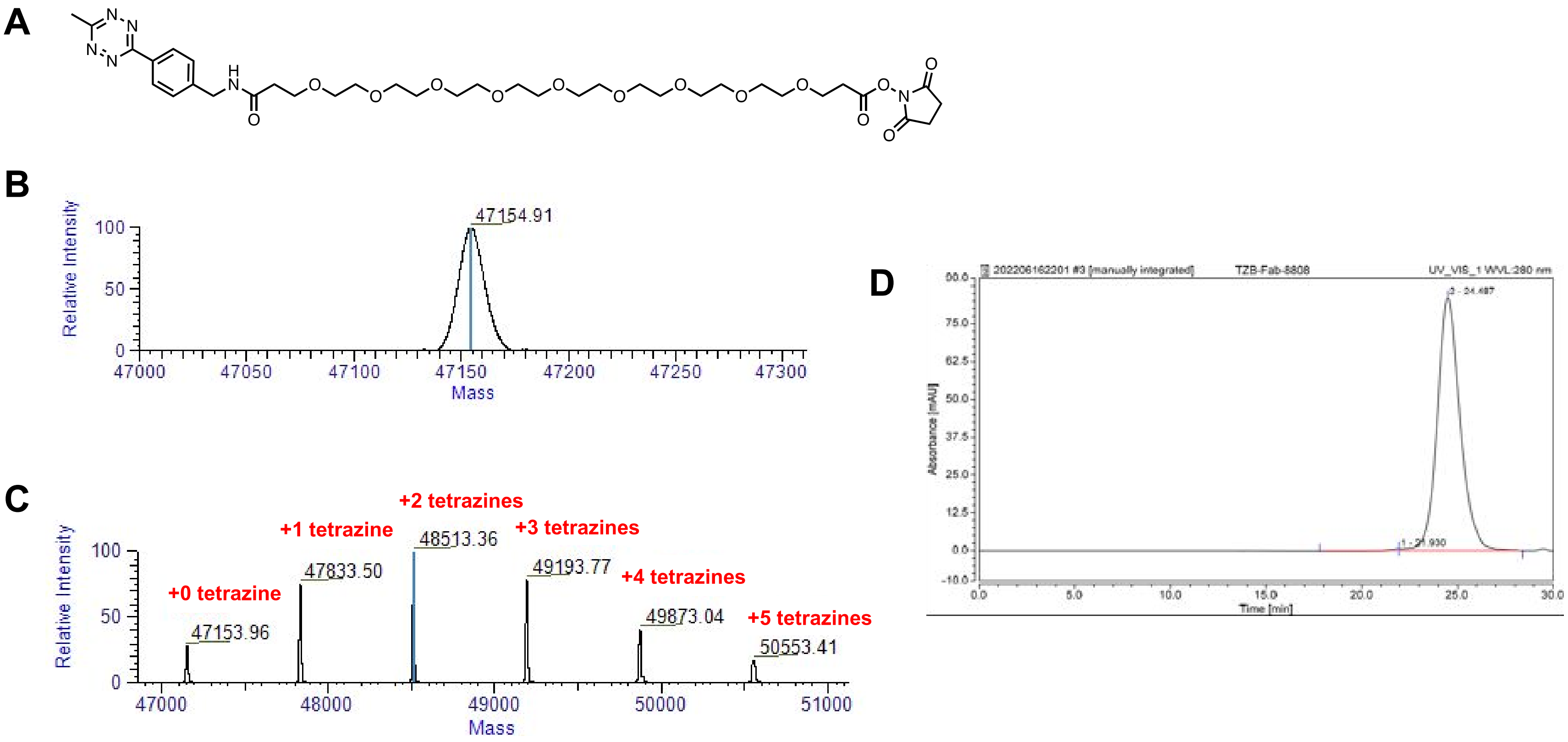

Figure S4. Characterization of SQT01. **A**, Structure of tetrazine-PEG9-NHS used to modify the Fab. **B**, Mass spectrum of unmodified Fab. **C**, Deconvoluted mass spectrum of tetrazine-modified Fab (calculated tetrazine-to-antibody ratio was 2.2). **D**, Size exclusion chromatography analysis showing > 99% monomeric species for the Fab-tetrazine conjugate. Fab, antigen-binding fragment; see Figure S18 for unprocessed mass spectra.

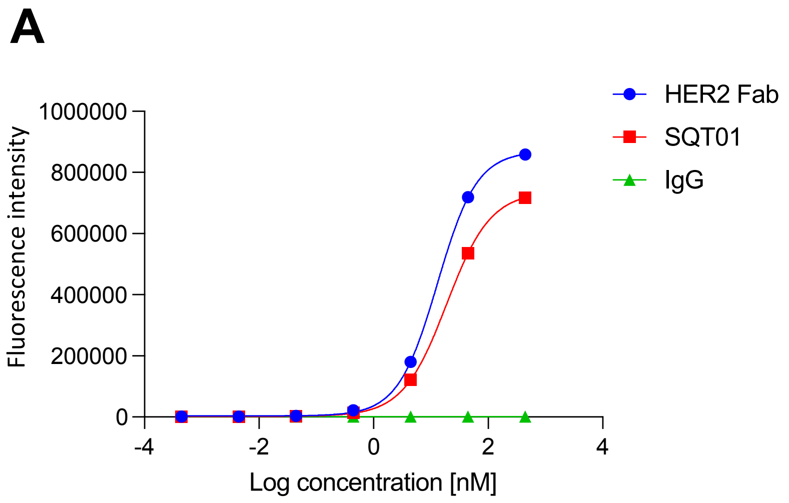

NCI-N87 cells		
Antibody (Ab) ID	max MFI	EC50 (nM)
HER2 Fab	869,554	13
SQT01	737,310	18.86
IgG	~ 1,141	/

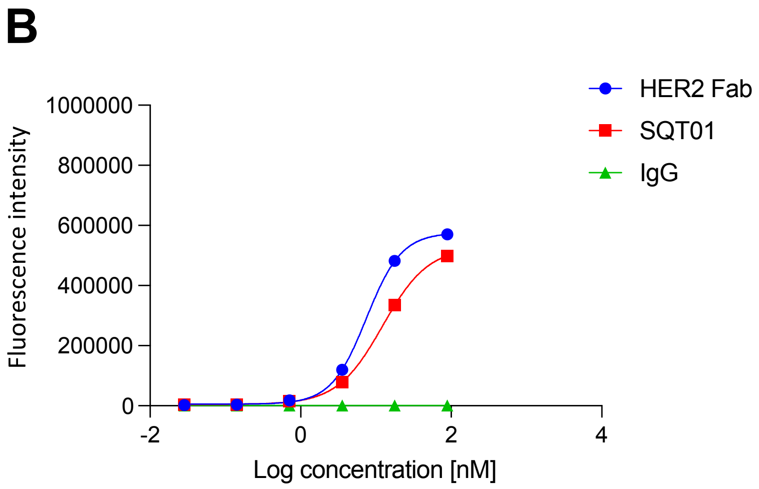

NCI-N87 cells		
Antibody (Ab) ID	max MFI	EC50 (nM)
HER2 Fab	576301	7.460
SQT01	527765	12.29
IgG	~ 1,146	/

Figure S5. Characterization of SQT01 binding. **A, B**, Separate experiments of binding of NCI-N87 cells by HER2 Fab, SQT01, and non-binding IgG control quantified by flow cytometry. Fab, antigen-binding fragment; HER2, human epidermal growth factor receptor 2.

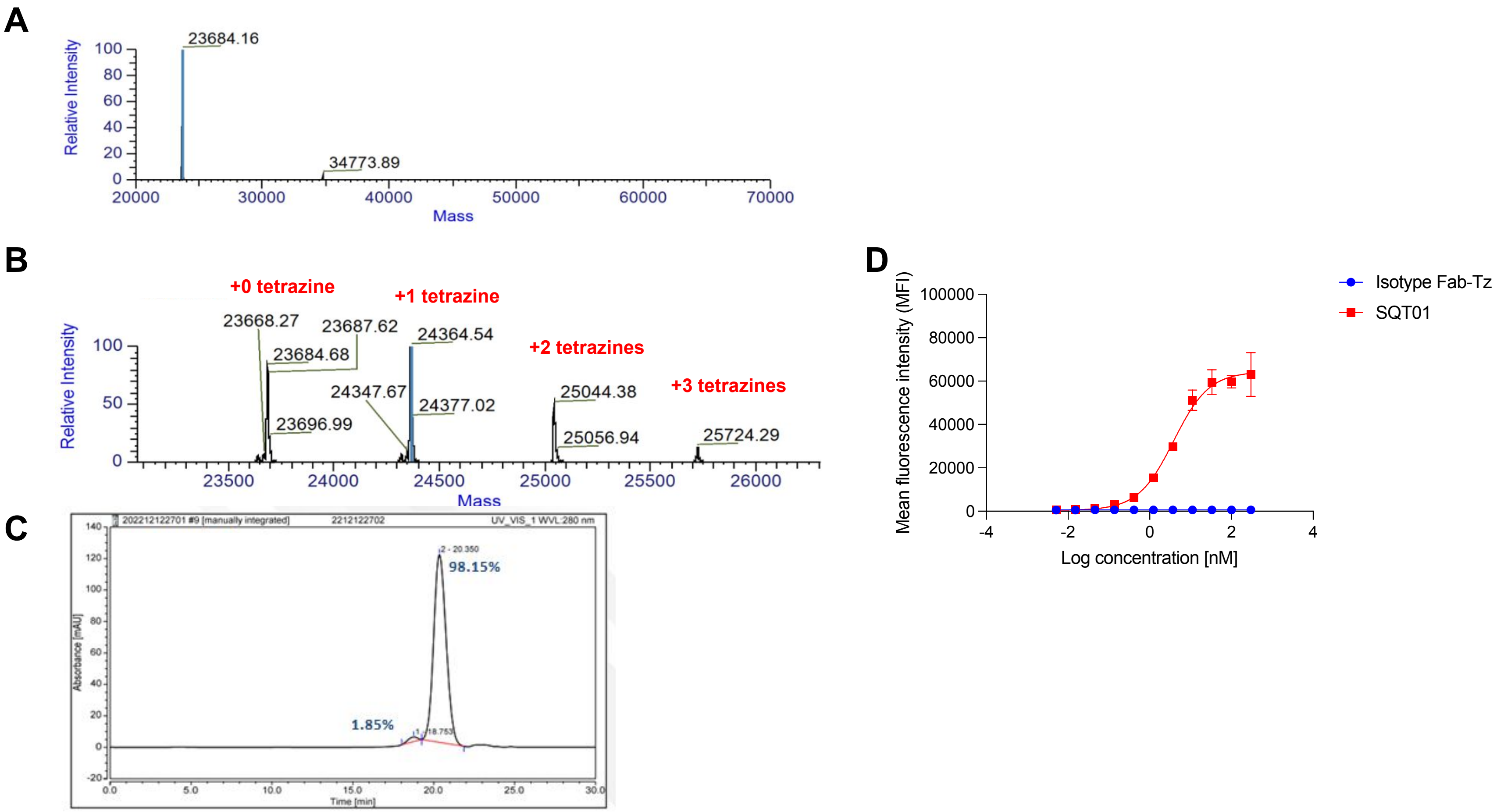

Figure S6. Characterization of Isotype Fab-Tz. **A**, Mass spectrum of unmodified Fab. **B**, Deconvoluted mass spectrum of tetrazine-modified Fab (calculated tetrazine-to-antibody ratio was 1.8). **C**, Size exclusion chromatography analysis showing > 99% monomeric species for the Fab-tetrazine conjugate. **D**, Isotype Fab-Tz does not bind NCI-N87 cells up at concentrations up to 300 nM as quantified by flow cytometry. Data shown as mean ± SEM (two technical replicates). Fab, antigen-binding fragment; see Figure S19 for unprocessed mass spectra.

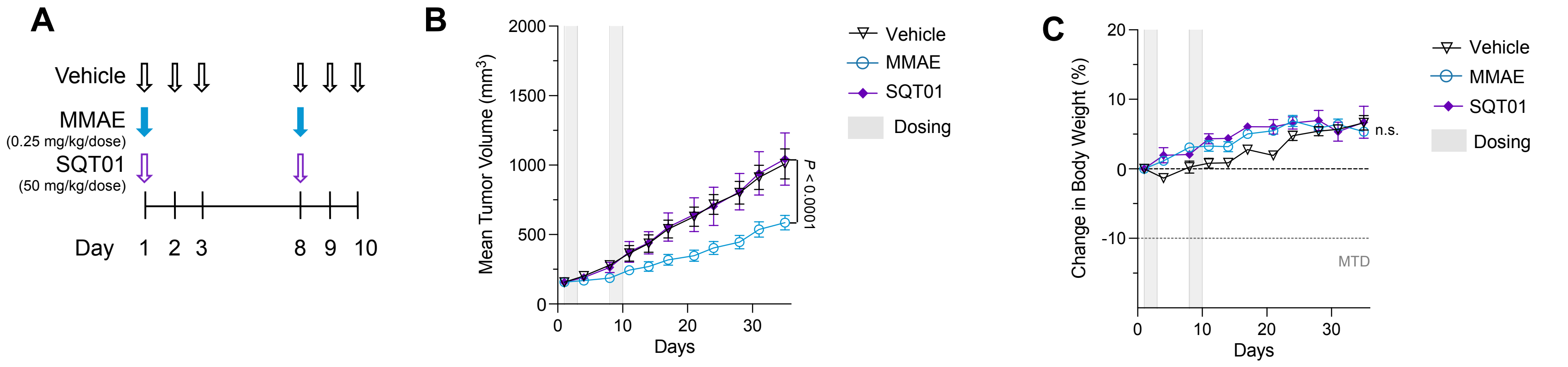

Figure S7. SQT01 has no effect on tumor volume or mouse body weight. **A**, Schedule of dosing of agents. **B**, Tumor volumes of NCI-N87 tumors in SCID mice treated with vehicle (n = 10 mice), MMAE (n = 10 mice), and SQT01 (n = 5 mice) in absence of a protodrug. **C**, Percent body weight change for the groups. Shown are means ± SEM. P-values were determined by two-way ANOVA with Bonferroni correction for multiple comparisons. MMAE, monomethyl auristatin E; MTD, maximum tolerated dose; n.s., not significant.

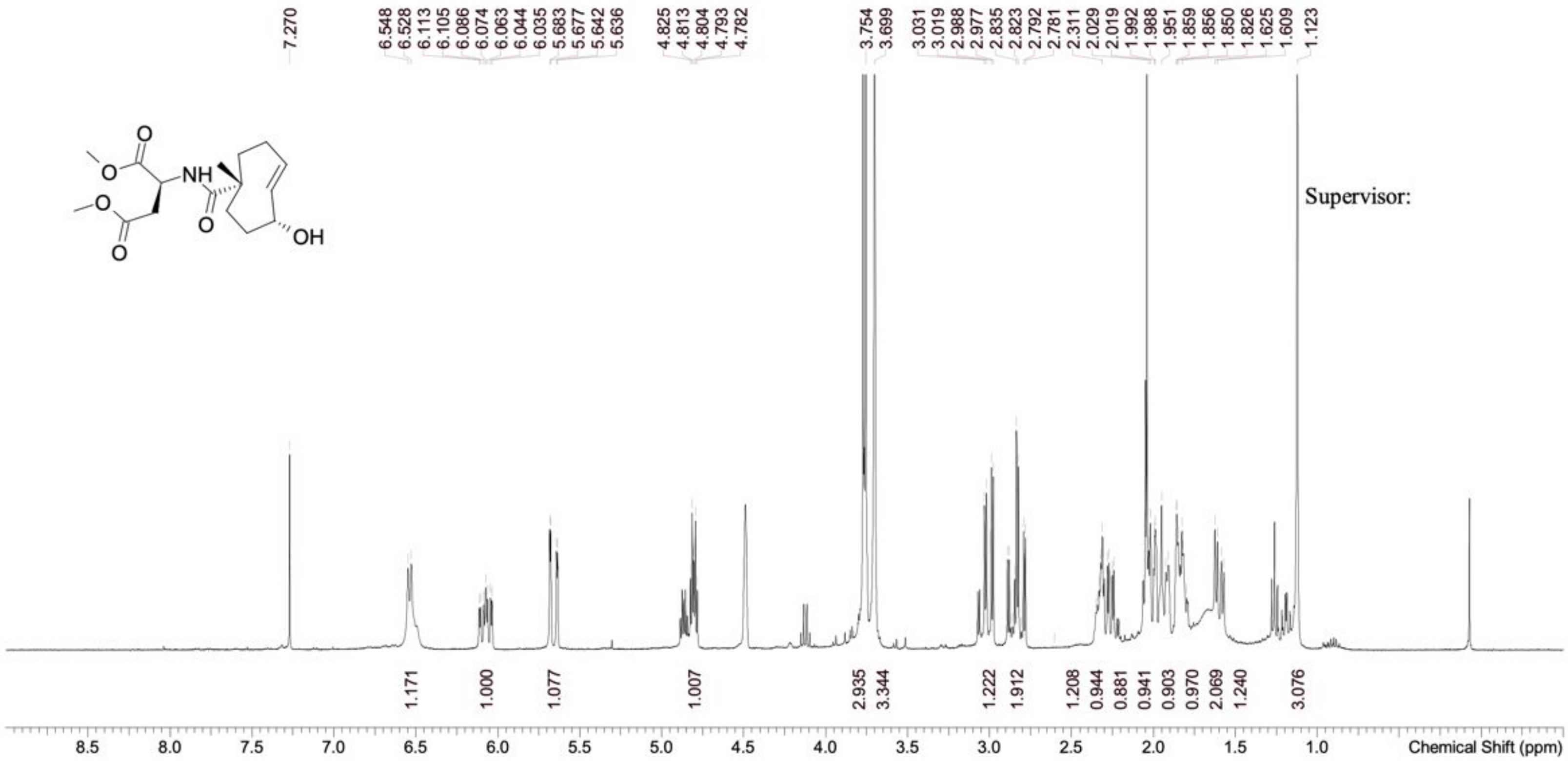

Figure S8. ^{1}H NMR of Compound **4**. ^{1}H NMR, proton nuclear magnetic resonance.

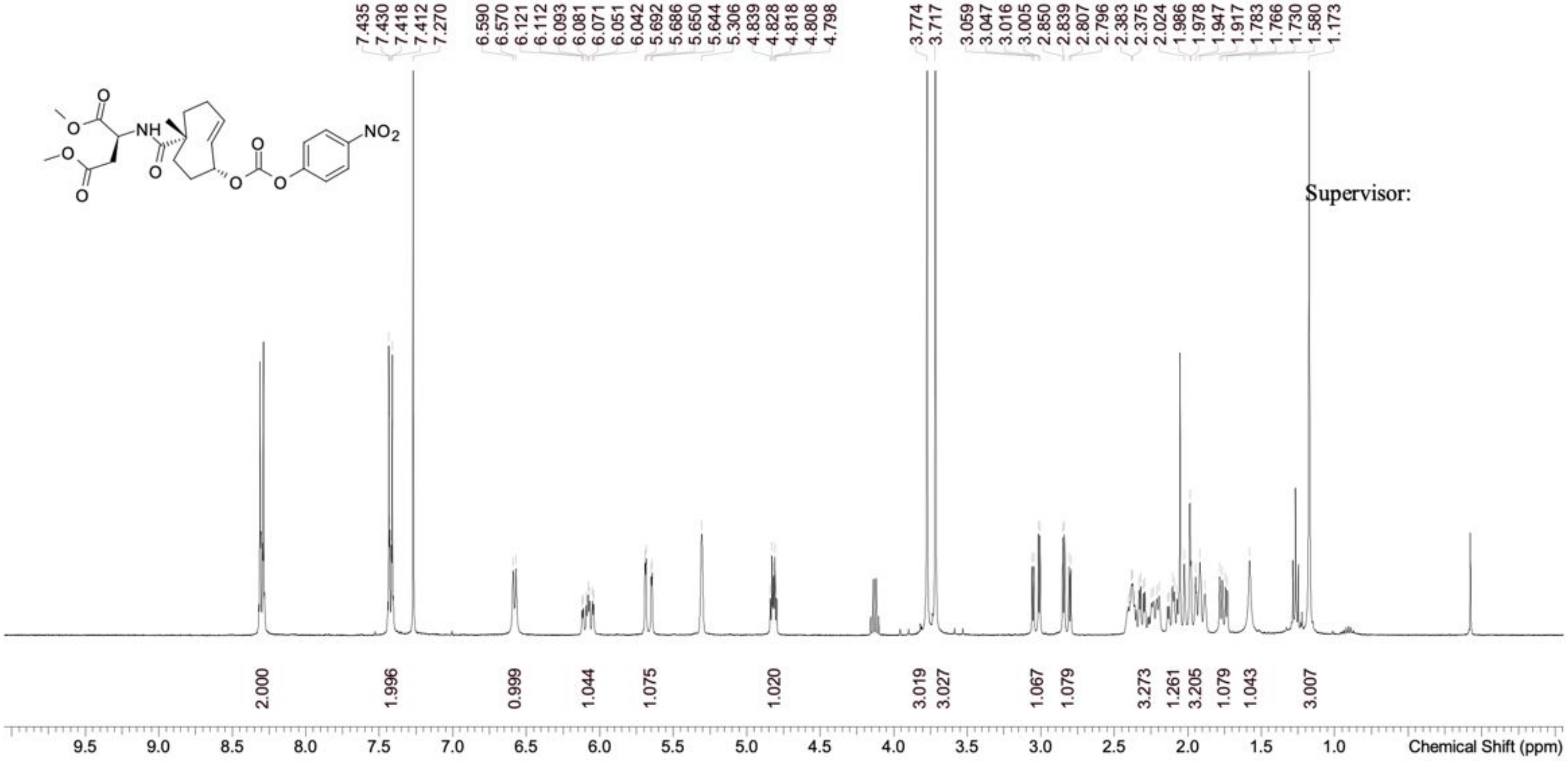

Figure S9. ^{1}H NMR of Compound **6**. ^{1}H NMR, proton nuclear magnetic resonance.

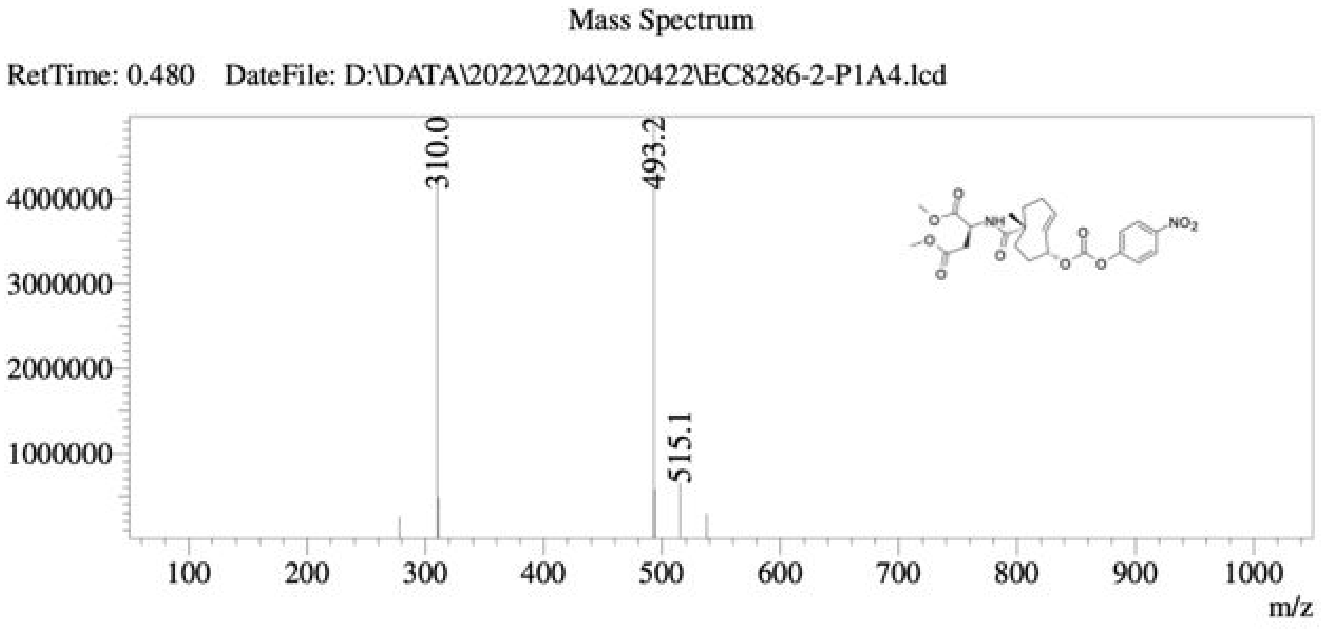

Figure S10. ESI-MS of Compound **6**. ESI-MS, electrospray ionization-mass spectrometry.

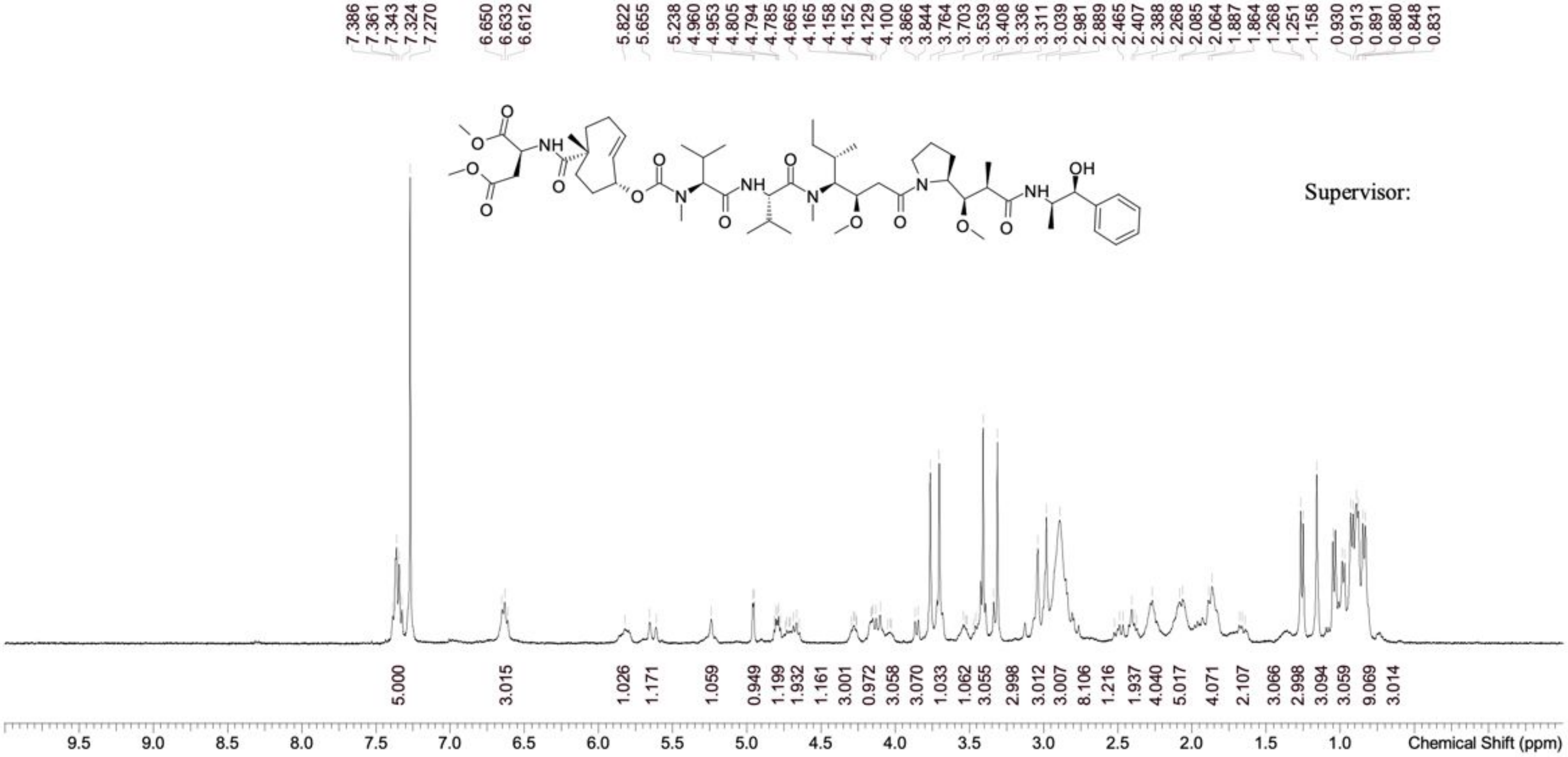

Figure S11. ^{1}H NMR of Compound **7**. ^{1}H NMR, proton nuclear magnetic resonance.

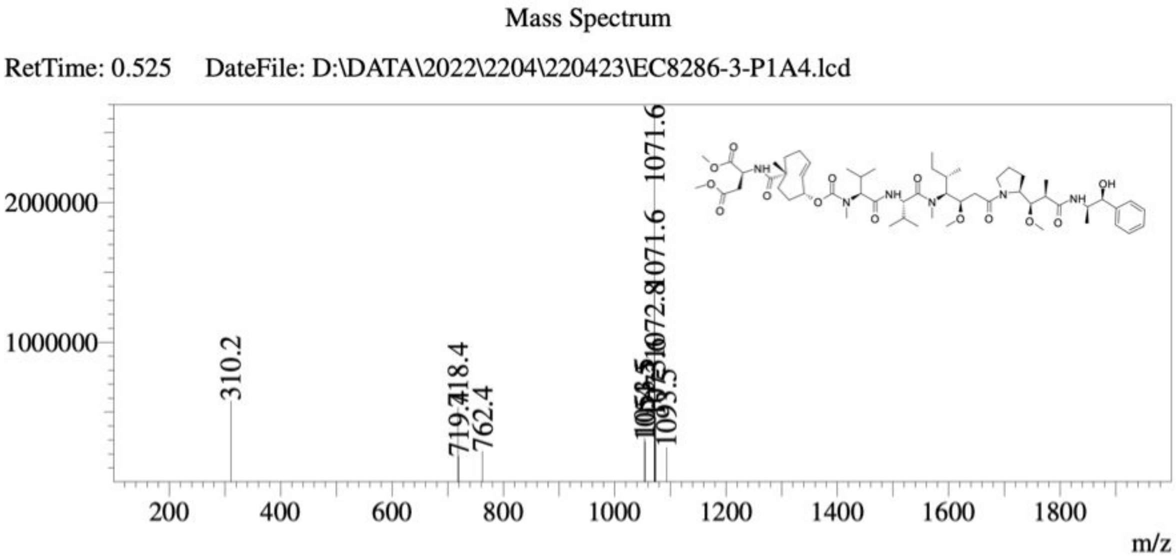

Figure S12. ESI-MS of Compound **7**. ESI-MS, electrospray ionization-mass spectrometry.

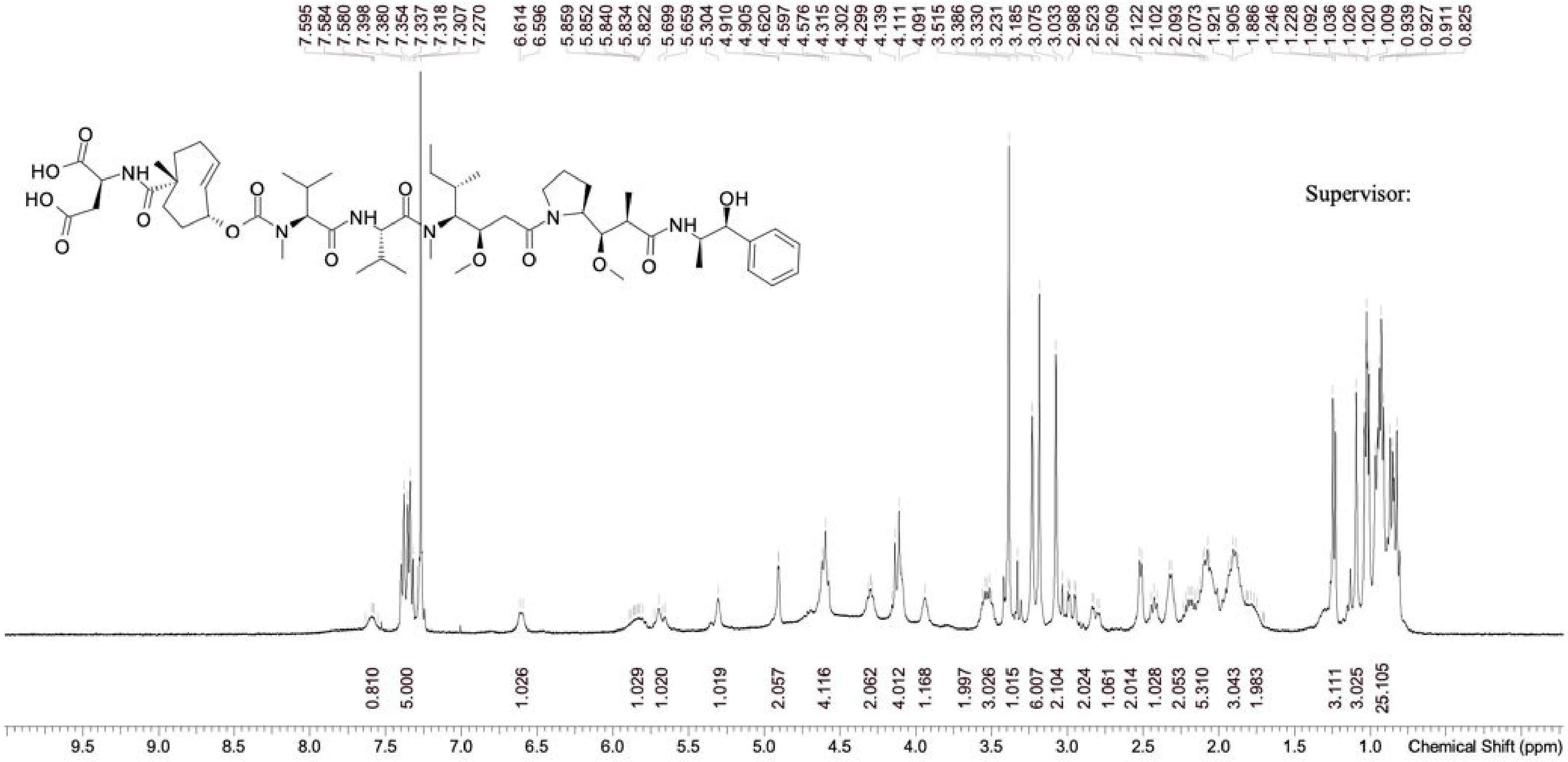

Figure S13. ^{1}H NMR of Compound **SQP22**. ^{1}H NMR, proton nuclear magnetic resonance.

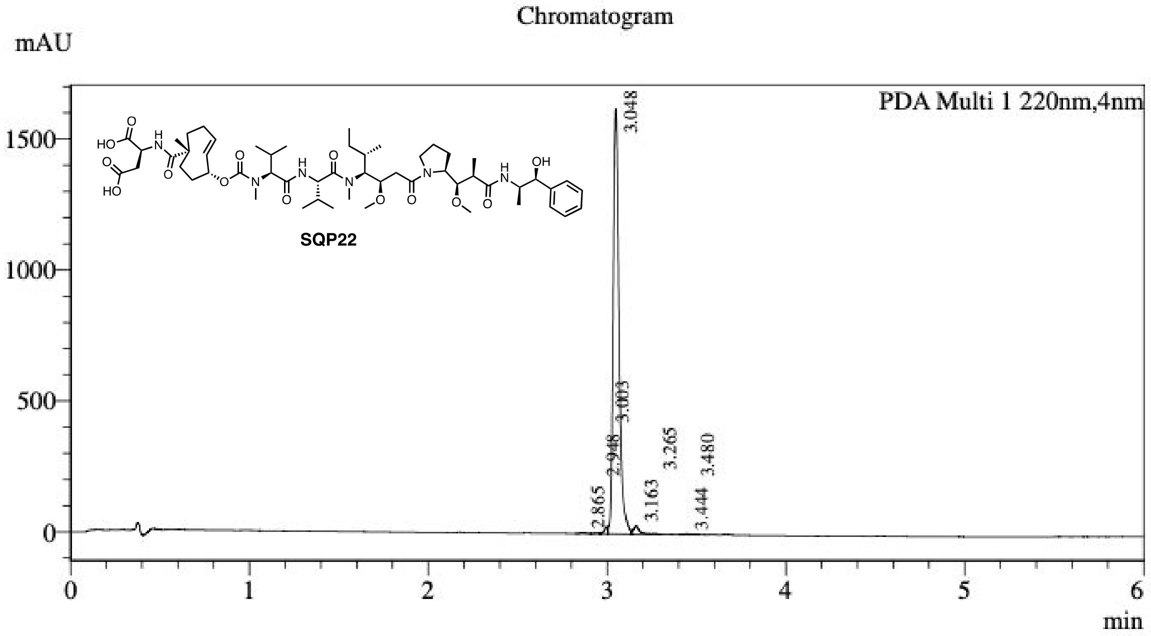

Integration Result

PDA Ch1 220nm

Peak#	Ret. Time	USP Width	Height	Height%	Area	Area%
1	2.865	0.055	3205	0.189	6939	0.182
2	2.948	0.096	2735	0.161	5111	0.134
3	3.003	0.443	27258	1.609	35708	0.937
4	3.048	0.057	1622601	95.779	3678315	96.518
5	3.163	0.058	29721	1.754	68297	1.792
6	3.265	0.053	2935	0.173	5604	0.147
7	3.444	0.251	3207	0.189	6695	0.176
8	3.480	0.286	2444	0.144	4349	0.114
Total			1694107	100.000	3811018	100.000

Figure S14. HPLC analysis of SQP22 (absorbance at 220 nm). Note MMAE retention time is 2.1 min (Figure S17). HPLC, high performance liquid chromatography; MMAE, monomethyl auristatin E.

Mass Spectrum

RetTime: 0.475 DateFile: D:\DATA\2022\2205\220511\EC8286-4-P1AA.lcd

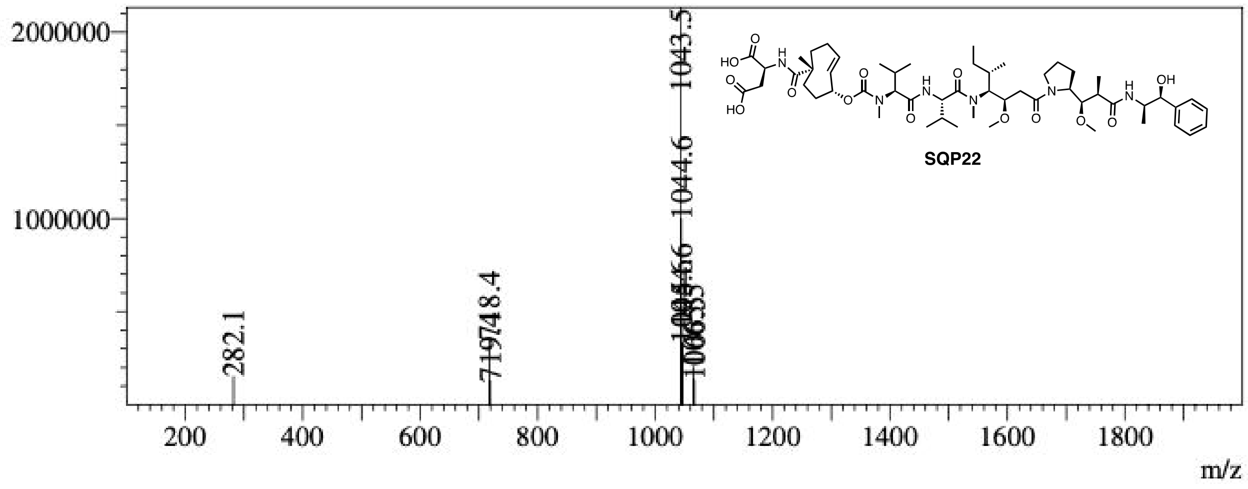

Figure S15. ESI-MS of SQP22. ESI-MS, electrospray ionization-mass spectrometry.

HRMS Report

Sample Information

**Sample ID**	EC8286-4-P1	**Data File Path**	D:\data\2022\202205\0512\EC8286-4-P1.d
**Compound ID**	Target 1-SQRD-015	**Acq. Time (Local)**	5/12/2022 11:49:12 (UTC+08:00)
**Instrument**	CAS-CD-QTOF-A	**Method Path (Acq)**	D:\data\methods\WUXIAB10_Aq_200V_100.m
**MS Type**	QTOF	**Operator**	pharmatechs.com\liu_ting3201
**Inj. Vol. (ul)**	0.1	**Position**	P1-A8

Sample Chromatograms

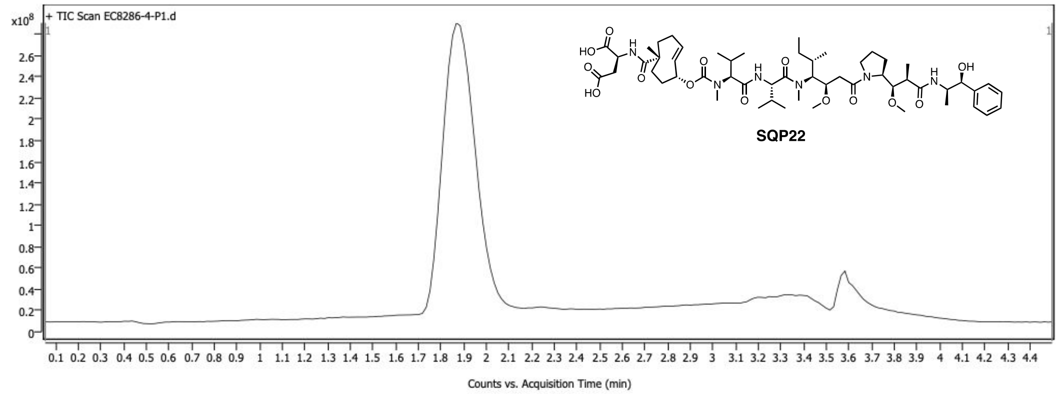

Compound Summary

Cpd	Name	Formula	CAS	RT	Mass	Mass (Tgt)	Diff (Tgt, ppm)	Score	Algorithm
1		C54 H86 N6 O14		1.868	1042.6203	1042.6202	0.12	99.66	FBF

Compound Details

Cpd. 1: C54 H86 N6 O14

Name	Formula	RT	RI	Mass	Diff (Tgt, ppm)	CAS	ID Source	Score	Algorithm
	C54 H86 N6 O14	1.868		1042.6203	0.12		FBF	99.66	FBF

Species	m/z	Score (Tgt)	Score (Lib)	Score (DB)	Score (MFG)	Score (RT)
(M+H)+ (M+Na)+	1043.6279 1065.6097	99.66				

Compound Chromatograms (overlaid)

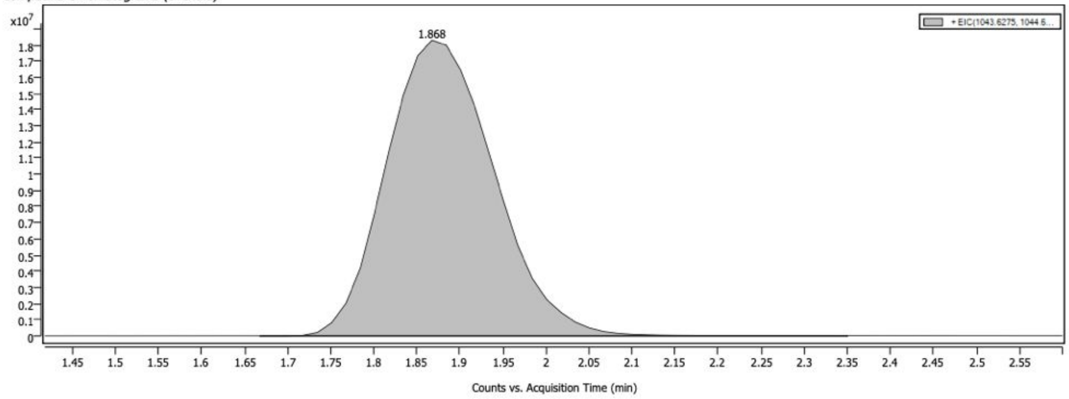

Compound Spectra (overlaid)

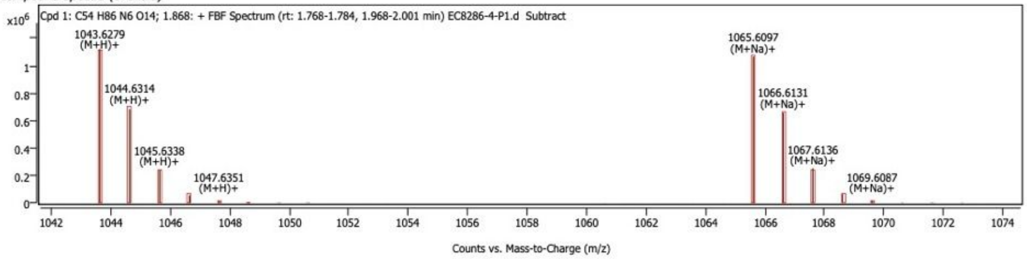

Compound ID Table

Name	Formula	Species	RT	RT Diff	Mass	CAS	ID Source	Score	Score (Lib)	Score (Tgt)
	C54 H86 N6 O14	(M+H)+ (M+Na)+	1.868		1042.6203		FBF	99.66		99.66

Figure S16. High resolution ESI-MS of SQP22. ESI-MS, electrospray ionization-mass spectrometry.

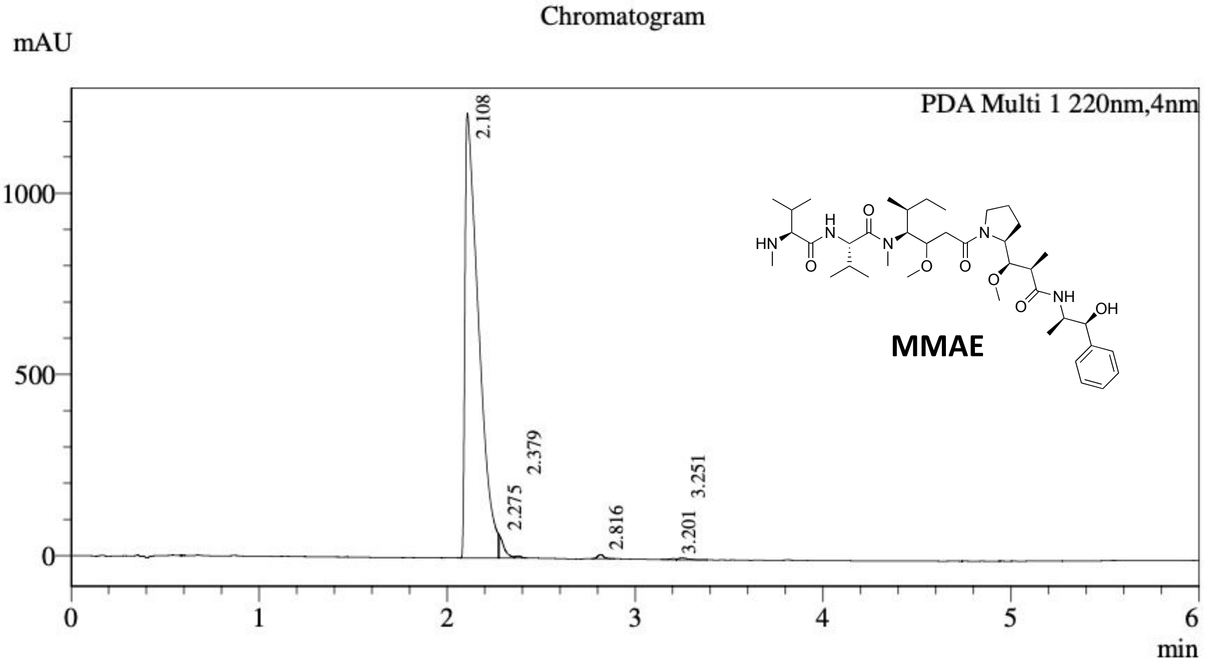

Integration Result

PDA Ch1 220nm

Peak#	Ret. Time	USP Width	Height	Height%	Area	Area%
1	2.108	0.142	1229450	93.207	6175701	97.153
2	2.275	0.047	64687	4.904	118617	1.866
3	2.379	0.073	5805	0.440	12496	0.197
4	2.816	0.057	11337	0.859	25307	0.398
5	3.201	0.208	3143	0.238	6749	0.106
6	3.251	0.109	4638	0.352	17776	0.280
Total			1319060	100.000	6356646	100.000

Figure S17. HPLC analysis of MMAE (absorbance at 220 nm). HPLC, high performance liquid chromatography; MMAE, monomethyl auristatin E.

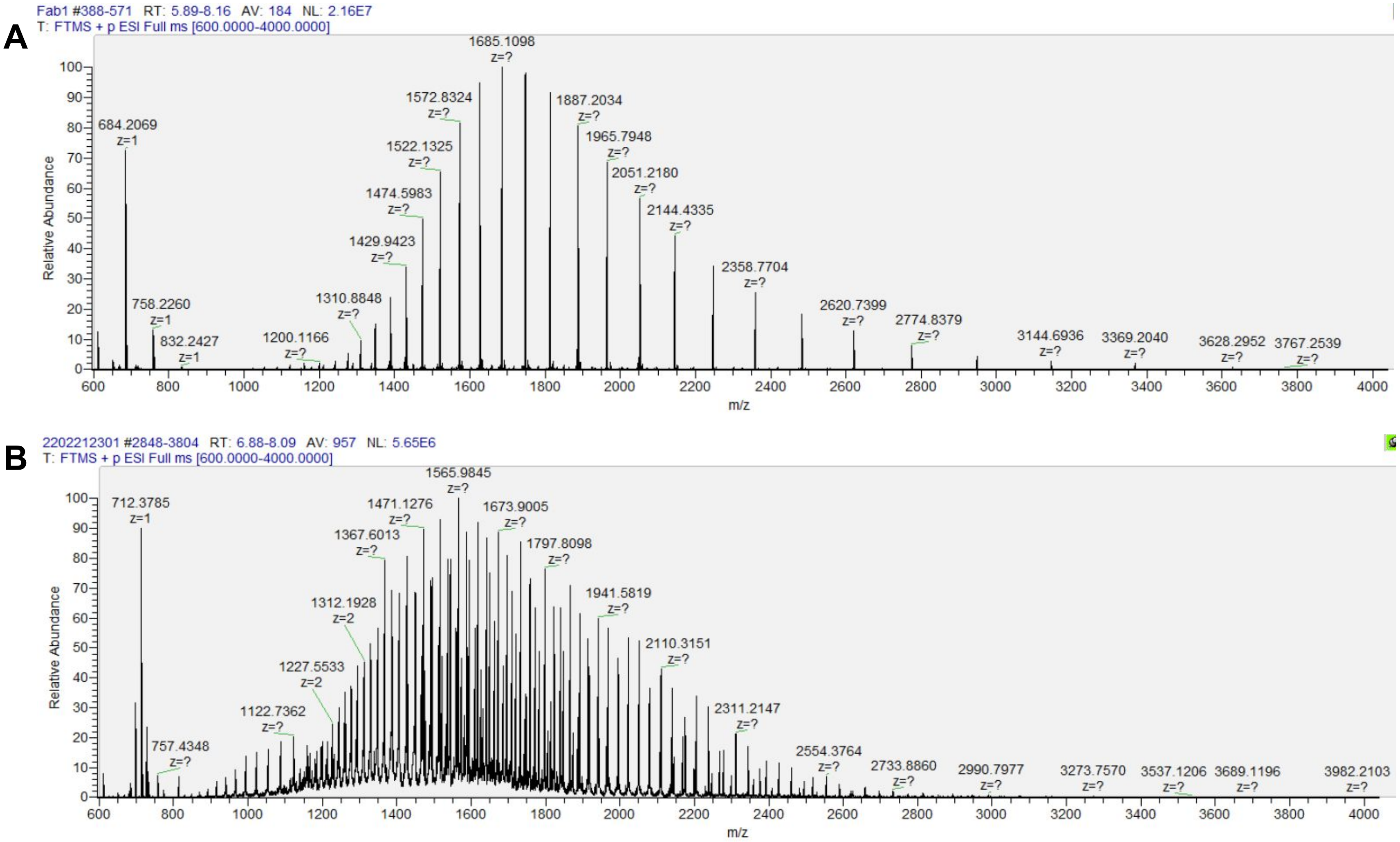

Figure S18. Non-deconvoluted ESI-MS of (A) HER2 Fab and (B) SQT01 conjugate. ESI-LCMS, electrospray ionization-liquid chromatography mass spectrometry; Fab, antigen-binding fragment; HER2, human epidermal growth factor receptor 2.

A

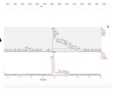

B

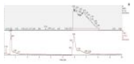

<u>Figure S19.</u> Non-deconvoluted ESI-MS of (A) Isotype Fab and (B) Isotype Fab-Tz conjugate. ESI-LCMS, electrospray ionization-liquid chromatography mass spectrometry. Fab, antigen-binding fragment; Fab-Tz; Fab, antigen-binding fragment tetrazine conjugate.
